# Stiffness transitions in new walls post-cell division differ between *Marchantia polymorpha* gemmae and *Arabidopsis* thaliana leaves

**DOI:** 10.1073/pnas.2302985120

**Published:** 2023-10-02

**Authors:** Alessandra Bonfanti, Euan Thomas Smithers, Matthieu Bourdon, Alex Guyon, Philip Carella, Ross Carter, Raymond Wightman, Sebastian Schornack, Henrik Jönsson, Sarah Robinson

**Affiliations:** ^a^Sainsbury Laboratory Cambridge University, Cambridge CB2 1LR, United Kingdom; ^b^Department of Civil and Environmental Engineering, Politecnico di Milano, Milan 20133, Italy; ^c^Cell and Developmental Biology, John Innes Centre, Norwich NR4 7UH, United Kingdom; ^d^Department of Applied Mathematics and Theoretical Physics, University of Cambridge, Cambridge CB3 0WA, United Kingdom; ^e^Department of Astronomy and Theoretical Physics, Computational Biology and Biological Physics, Lund University, Lund 223 62, Sweden

**Keywords:** *Marchantia polymorpha*, cell division, atomic force microscopy, biomechanics, modeling

## Abstract

Plant morphogenesis is governed by the mechanics of the cell wall—a stiff and thin polymeric box that encloses the cells. The cell wall is a highly dynamic composite material. New cell walls are added during cell division. As the cells continue to grow, the properties of cell walls are modulated to undergo significant changes in shape and size without breakage. Spatial and temporal variations in cell wall mechanical properties have been observed. However, how they relate to cell division remains an outstanding question. Here, we combine time-lapse imaging with local mechanical measurements via atomic force microscopy to systematically map the cell wall’s age and growth, with their stiffness. We make use of two systems, *Marchantia polymorpha* gemmae, and *Arabidopsis thaliana* leaves. We first characterize the growth and cell division of *M. polymorpha* gemmae. We then demonstrate that cell division in *M. polymorpha* gemmae results in the generation of a temporary stiffer and slower-growing new wall. In contrast, this transient phenomenon is absent in *A. thaliana* leaves. We provide evidence that this different temporal behavior has a direct impact on the local cell geometry via changes in the junction angle. These results are expected to pave the way for developing more realistic plant morphogenetic models and to advance the study into the impact of cell division on tissue growth.

## Significance

During morphogenesis, plant cells divide and undergo significant shape changes. The mechanical properties of the cell wall during these two processes are important for plant morphogenesis. We introduce a systematic method to map cell wall age and growth to its bulk elasticity. We demonstrate that the stiffness of the wall correlates with their growth and that the new walls in *Marchantia polymorpha* gemmae become transiently stiffer and slower-growing compared to the older walls, a phenomenon not seen in *Arabidopsis thaliana* leaves. Using computational modeling, we were able to link the properties of the new walls to a local cell shape change in the two species—junction angle between the new and the old walls.

The development of multicellular tissues requires cell division, cell expansion, and differentiation. Compared to animal cells, plant cells are surrounded by a stiff polymeric structure that impedes the movement of cells (1), referred to as the cell wall (2). Therefore, the wide range of shapes observed in nature requires deformations and growth at the cell level of such rigid enclosing boxes (3). This aspect makes the understanding of the mechanical properties of the cell wall key for the study of plant morphogenesis. New wall material is deposited during cell division and, as the cells grow, cell walls are continuously remodeled. This remodeling process enables significant changes in shape while preventing breakage (4).

Plant cell walls loosen during cell expansion to accommodate large changes in their shape (3, 5) and area—often up to 100-fold (4, 6). It has been widely shown that different tissues (e.g., hypocotyl, leaf) do not grow isotropically, but they possess preferential growth directions (7, 8). This suggests that the mechanical properties of cell walls may not be homogeneous within a tissue. Macroscale tensile tests have shown that tissue growth inversely correlates with bulk stiffness (9–11). Local measurements of cell wall stiffness via atomic force microscopy (AFM) of shoot apical meristem have revealed that cell walls located at the tip are stiffer than those at the flanks (12, 13). This correlates with the growth analysis showing that cells at the tip of the meristem have slower growth with respect to those at the flanks (12, 14). Similarly, it has been shown experimentally that softening of the longitudinal anticlinal cell walls in hypocotyls (8) precedes its anisotropic growth, although this is unlikely to be sufficient without the contribution from the internal layers (15).

During cell division, new wall material is added within the tissue at a new location. Given the importance of cell division in morphogenesis, the prediction of the placement of the new wall has been extensively studied. With some notable exceptions—such as stomatal lineage cells (16, 17), the early embryo (18) and cambium differentiation (19, 20)—the division path follows either the statistical shortest path (21, 22) or the direction of maximal tensile stress (23, 24). Due to the prominent contribution of cellgeometry to cell stress, the direction of maximal tension in a cell often aligns with the shortest path (25), except in regions with high tissue stress, such as the boundary region of the shoot apical meristem (24).

The cell division process alters the mechanical properties of the tissue and in turn its growth (26). Recent research has revealed that in leaves, cell division occurring in the abaxial–adaxial plane plays a key role in preserving the flat shape of the organ (27). Computational models have shown that the addition of new walls following the shortest path or maximum stress direction locally decreases the stress in neighboring cells more than a random division orientation (28). When cell divisions follow the shortest path or maximum stress direction, the resulting lower stress in neighboring cells promotes a more uniform growth dynamics. Currently, in studies of plant morphogenesis, cell division is often modeled as the appearance of a new edge with either the same or stiffer properties than the surrounding ones (29, 30). This implementation is based on the assumption that cell wall deposition occurs instantaneously and that the new wall will possess the same or stiffer properties as the surrounding ones at the end of the division process.

The mechanical properties of cell walls are directly related to their geometry, composition, and architecture (9, 31, 32). In the well-established model system *A. thaliana*, it has been shown that, during cell division, callose deposition establishes the foundation for the future wall—known as the cell plate—followed by the gradual integration of various cell wall polymers (33, 34). Based on the changes in wall composition observed experimentally, a time evolution of the mechanical properties of the cell wall during cell division is expected. Yet, how the mechanical properties of the cell wall temporally change during cell division up until its mature stage remains understudied, as well as its local impact on the cell shape and tissue growth.

Addressing the spatial and temporal modulation of the mechanical properties of cell walls during cell division and cell expansion is paramount to advancing our understanding of plant morphogenesis. Here, we combine time course imaging of cells, with AFM measurements, to systematically map the age, growth, and mechanical properties (stiffness) of individual cell walls—thus, we perform an optomechanical coupling. We use this approach to address how the stiffness of newly formed cell walls varies in time and how this affects local cell shapes. To do so, we make use of two systems: the gemma of the liverwort *M. polymorpha*, and the leaves of the seed plant *A. thaliana*. *M. polymorpha* gemmae are clones of the mother plant and it is capable of growing into full plants into 3 wk (35, 36). Gemmae possess a flat sheet-like body (35) that can be easily imaged without dissection. Its flat configuration allows easy access to cell walls for on-contact mechanical testing like atomic force microscopy. This feature makes *M. polymorpha* gemmae an ideal model for this study. We first characterize the growth and cell division patterns in *M. polymorpha* gemmae, while those of *A. thaliana* leaves are already well established. The mechanical properties of new walls are then studied in both *M. polymorpha* gemmae and *A. thaliana* leaves. We show that cell division in *M. polymorpha* gemmae results in the generation of a temporary stiffer and slower growing new wall. In contrast, this transient phenomenon is absent in *A. thaliana* leaves. We show that this different temporal behavior directly impacts the local cell geometry—junction angle of the new wall with respect to the old wall. Additionally, we are able to demonstrate a correlation between the measured change in stiffness and the individual cell wall growth.

## Characterization of Growth and Cell Division in M. polymorpha Gemmae

**Fig. 1.**
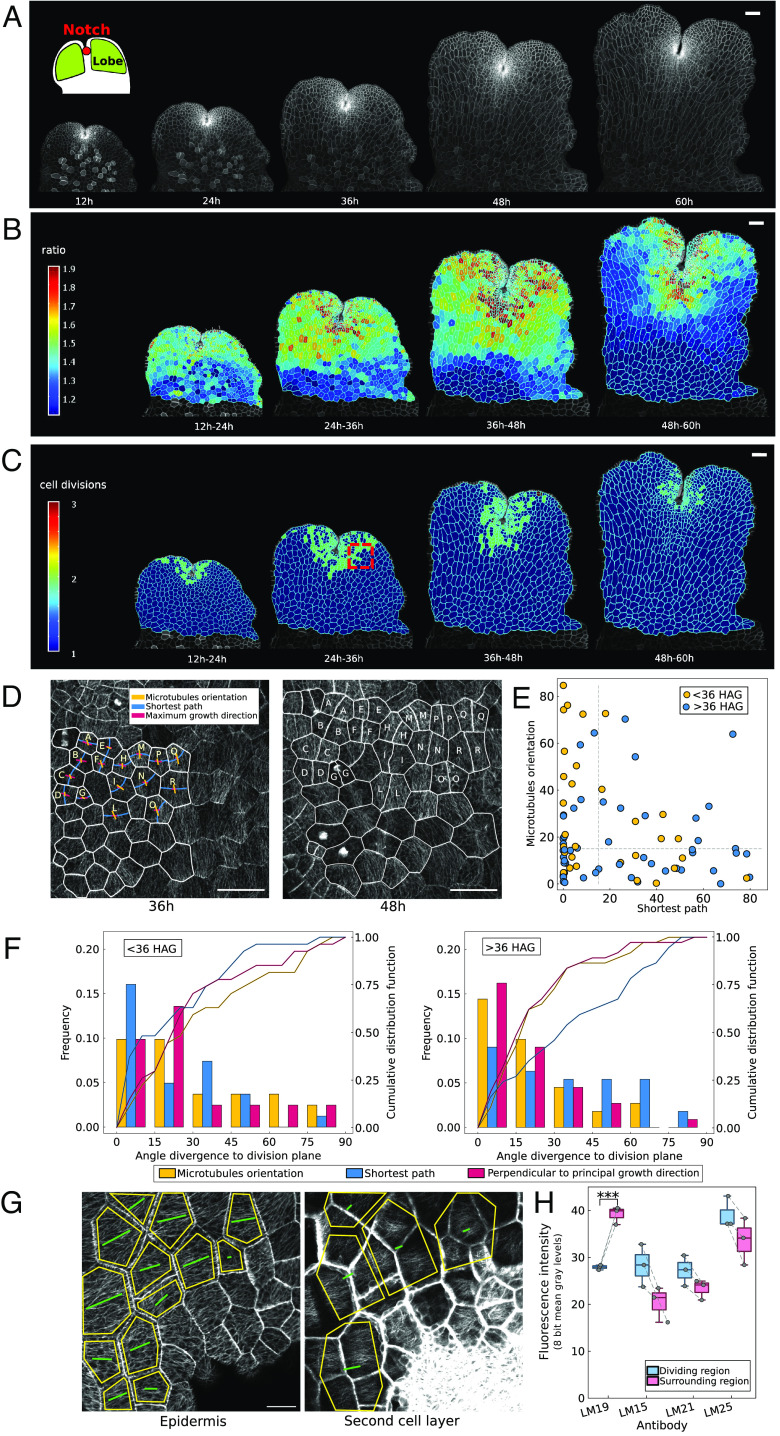
Quantification of growth and cell division in *M. polymorpha* gemma (*A*) Time-lapse confocal images of half a gemma every 12 h in hours after germination (HAG). (Scale bar, 100 μm.) (*B*) Heat map of the ratio of cell area over the 12-h time period shown in HAG. (Scale bar, 100 μm.) White lines show the major axis of expansion. (*C*) Heatmap of the number of cell divisions in the period of time shown. (Scale bar, 100 μm.) (*D*) Sequential imaging of cells cortical microtubules in the division zone (example location identified with the red square in *C*) from which we extracted: i) the main direction of microtubule orientation (yellow), ii) maximum growth direction (pink), iii) shortest division plane prediction (blue). (Scale bar, 50 μm.) (*E*) Plot of the angle between microtubule orientation and actual division orientation versus angle between the shortest path and actual division orientation for each cell division (<36 HAG, yellow dots; >36 HAG, blue dots). The dash lines are placed for reference at 15${}^{{}^{\circ}}$ used as the limit to compute microtubule orientation and shortest path mismatch (*Material and Methods*). (*F*) Quantification of angles between the actual division orientation and: i) microtubule main orientation (yellow), ii) the shortest path (blue), iii) direction perpendicular to the principal growth direction (pink)—total number of cells 64. The cells for which both microtubule orientation and shortest path possess an angle of divergence to division plane < 15° have been removed (full data *SI Appendix*, Fig. S1*D*). The first plot refers to the events occurring before 36 HAG (number of events = 27), the second plot refers to the events occurring after 36 HAG (number of events = 37). The yellow, blue, and pink solid lines represent, respectively, the cumulative distribution for the three predictions—microtubule orientation, shortest path, and PGD. A comparison of the cumulative distributions for the microtubule orientation and the shortest path shows that cell division in *M. polymorpha* gemmae better follows the shortest path for the events occurring before 36 HAG (Anderson–Darling test, *P* value = 0.0416), while for events 36 HAG the microtubule orientation is a better predictor (Anderson–Darling test, *P* value = 0.0247), which is a proxy for tension direction. The direction perpendicular to PGD shows good alignment with microtubule orientation and it thus is also a good predictor for division orientation after 36 HAG (*P* value between perpendicular to PGD and microtubule orientation: <36H = 0.83 n.s, >36HAG= 0.96 n.s.- Anderson–Darling test). (*G*) Microtubule orientation in the outer cell layer—epidermis—matches that of the top cell face of the second cell layer, 36 HAG. (Scale bar, 20 μm.) The green lines show the main directions of microtubule orientation as computed via FibrilTool, while the yellow contours depict the areas considered for the quantification. (*H*) Comparison of the different antibodies intensity in *M. polymorpha* gemmae in the highly dividing meristem area (dividing region) and in the surrounding area. The change in intensity of the LM19—unesterified homogalacturonan—is significant (*t* test *P* value = 0.0006, Wilcoxon rank sum test), while all the other changes result nonsignificant (*P* values: LM15 = 0.079, LM21 = 0.157, LM25 = 0.191, all n.s. Wilcoxon rank sum test). The gray dash lines connect the points belonging to the same sample.

The flat gemmae of *M. polymorpha* possess smooth, sometimes undulating edges and deep invaginations, referred to as notches. Macroscopic-scale tracking of air pores has shown that tissue growth occurs predominantly within the region surrounding the notch (37) (Fig. 1*A*, *Graphical Inset*). Here, we performed time-lapse imaging on early *M. polymorpha* gemmae to quantify their development at the cellular scale (Fig. 1*A*). We followed *M. polymorpha* gemmae development for 60 h, with 3-h intervals—plotted in Fig. 1*A*–*C* every 12 h to facilitate graphical representation. Gemmae possess a symmetric shape with respect to their central axis (*SI Appendix*, Fig. S1*A*). An initial time course of the whole gemmae (*SI Appendix*, Fig. S1 *B* and *C*) confirmed that the growth and cell division patterns are symmetric with respect to the central axis; subsequent characterization, therefore, focused on half of the gemma (Fig. 1*A*–*C*). Gemmae start to germinate once they are removed from gemma cups (38), thus allowing the synchronization of all the experiments by setting time zero as the removal of the gemmae from the cups—referred to hereafter as germination. Quantification of cell growth using MorphoGraphX (39, 40) showed that the slowest growth occurred in the central region of the gemmae, while the highest areal expansion occurred at the area surrounding the notch and within the lobes (Fig. 1*B*). Note that the outermost layer of cells was excluded from the segmentation as it was difficult to identify the outer edge, resulting in inaccurate area and growth calculations. The cells of the gemmae, and in turn the gemmae as a whole, expand anisotropically to create an elongated shape from initially more rounded gemmae (see white lines in Fig. 1*B* indicating the principal growth direction of each cell). Areal expansion is highest in the period 24 to 36 hour after germination (HAG).

Quantification of cell division frequency (Fig. 1*C*) confirmed that cell division is highest at a restricted area surrounding the notch. To determine what controls cell division orientation during early gemma development, enlarged time-lapse imaging of cells located at the meristem was performed for both the plasma-membrane and microtubule signals. We computed the shortest path connecting two nonconsecutive walls of dividing cells extracted from 48-h-long time lapse with a 12-h imaging interval (*Materials and Methods*). Cell geometry was extracted from the plasma-membrane signal via image segmentation using MorphoGraphX. Further, we computed the Principal Growth Direction (PGD) for the dividing cells using the segmented images via MorphoGraphX. At the same time, the direction of maximum tissue stress was approximated using the orientation of the microtubules (41–43) using FibrilTool (44) (Fig. 1*D*). For 30% of the total cell division events, the shortest path prediction and the major orientation of microtubules both aligned with the actual plane of division—the divergence angle for both the predictions with the actual plane of division was below 15° (*SI Appendix*, Fig. S1*D*). For the subsequent analysis, we only consider the division events for which the shortest path and microtubule orientation prediction did not match. By plotting the divergence angle between the observed cell division orientation and both the shortest path as well as the major microtubule orientation in relation to hours after germination (<36 HAG and >36 HAG), we identified a temporal shift in the influential factor governing cell division (Fig. 1*E*)—shortest path or microtubule orientation. Therefore, a more in-depth analysis was performed. We plotted the frequency distribution of the divergence angle between the actual plane of division and: i) shortest path and ii) microtubule orientation. This analysis was performed for division events occurring before 36 HAG and after 36 HAG. We found that before 36 HAG, cell geometry was a better predictor of cell division orientation than microtubule orientation (Fig. 1*F*, *Left*). The opposite was true for division events occurring later than 36 HAG (Fig. 1*F*, *Right*). Therefore, cell division orientation in *M. polymorpha* gemmae is more influenced by geometrical clues at early time points (<36 HAG). At later time points (>36 HAG), microtubules are a better predictor of cell division orientation, suggesting that geometrical clues may be overruled by tissue-level stresses. This result is further supported by recent work to quantify cell division in the notch region (45) that shows a correlation between cell division orientation and microtubule orientation during the study of ROP signaling in *M. polymorpha* gemmae. Consistent with the role of microtubules in influencing PGD (i.e., microtubules are perpendicular to PGD) via orienting cellulose deposition (46, 47), cell division orientation was comparably predicted by microtubule orientation or the direction perpendicular to the PGD, (Fig. 1*F*), thus being more accurate for events occurring after 36 HAG. The tissue-level stress results from the cell’s turgor pressure, the force exerted by the growing inner layers on the epidermal cells, and the shape of the tissue. Imaging of the internal layers (L2), showed good alignment between microtubule orientation in the L1 and L2 layers (Fig. 1*G*). This finding suggests the existence of a directed stress arising from the inner cells that is transmitted to the epidermal cells. It has been observed previously in *A. thaliana* hypocotyls that the inner layers have highly aligned microtubules (43, 48) and exert a stress on the outer layers (15, 49, 50).

To examine whether there is a difference in cell wall composition between the rapidly dividing region and the area within the lobes of *M. polymorpha* gemmae, we initially tested the effectiveness of primary cell wall probes that are well established in *A. thaliana* on *M. polymorpha* cell walls. It is well known that cell expansion in *A. thaliana* is regulated by the pectin methyl-esterification levels (51). In thin cross-sections of gemmae, the antibody specific for unesterified homogalacturonan [LM19 (52)] labeled all cell walls and vertices, whereas the antibody specific for methyl-esterified homogalacturonan [LM20 (52)] yielded very low signal (*SI Appendix*, Fig. S2). This suggests either LM20 epitopes are masked by another wall component or gemmae cell wall homogalacturonan has a low degree of methyl-esterification. Other well-known cell wall probes previously reported in *A. thaliana* studies also labeled both cell edge and vertices in *M. polymorpha* gemmae probes being the hemicelluloses xyloglucans [LM15 (53), LM25 (54)] and heteromannans [LM21 (53)] (*SI Appendix*, Fig. S2). We used the selected antibodies (LM19, LM15, LM25, and LM21) that successfully labeled cell walls in *M. polymorpha* gemmae to compare the cell wall composition of walls within the highly dividing region versus those in the lobes—where division rarely occurs. When comparing the relative fluorescence intensities obtained with the selected epitopes between the highly dividing region and the surrounding tissue in the gemmae, we observed changes. A significant difference was observed with the LM19 antibody (Fig. 1*H*), showing weaker fluorescence in the highly dividing region compared to the surrounding tissue (immunostaining sections for LM19 are shown in *SI Appendix*, Fig. S3). Our results suggest that some cell wall properties may be different in *M. polymorpha* gemmae compared to *A. thaliana* (51), and that levels of unesterified homogalacturonan are lower in the dividing region versus the nondividing region.

## Cell Division Generates Cell Walls with Transiently Different Mechanical Properties in M. polymorpha Gemmae

**Fig. 2.**
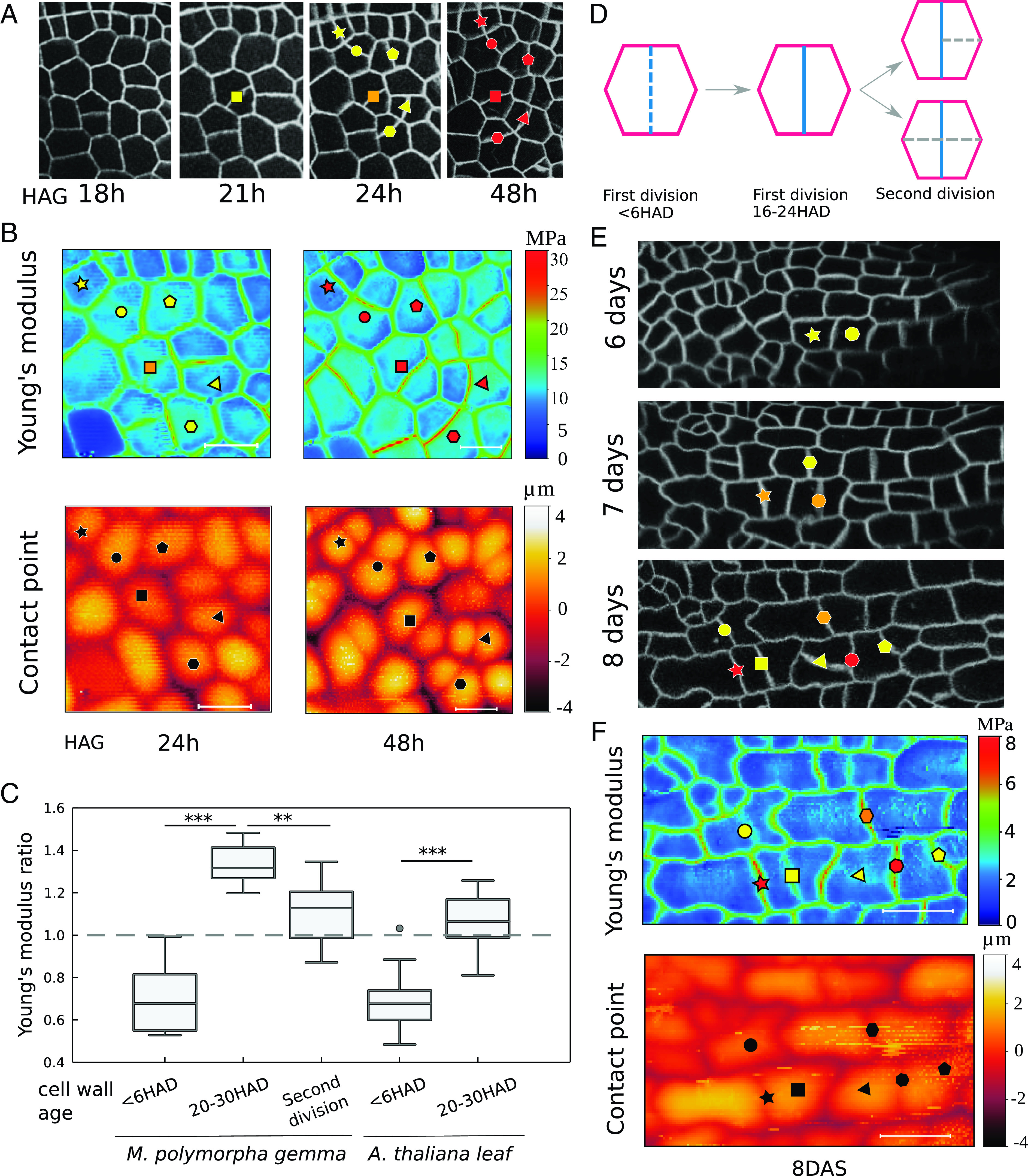
New cell walls show different mechanical dynamics in *M. polymorpha* and *A. thaliana*. (*A*) A patch of cells in *M. polymorpha* gemma is followed by time-lapse confocal imaging (from 18 HAG to 48 HAG). The individual newly formed walls are identified via color-coded markers across time points (< 6 h—yellow, 20 h>—red). (*B*) Atomic force microscopy time course on the imaged cells. Plots of the stiffness and contact point maps for the dividing cells at different HAD (24 HAD and 48 HAD) for *M. polymorpha*. (Scale bar, 20 μm.) The contact point map shows the change of the height of the new wall relative to the mother anticlinal wall. (*C*) Plot of Young’s modulus ratio against the new cell walls’ age (HAD). Young’s modulus ratio is the ratio between the apparent Young’s modulus of the new cell wall and the average apparent Young’s modulus of the surrounding walls. The boxplot shows that the stiffness of newly formed walls (<6 HAD) is lower than the surrounding walls of the mother cell. This is true for both *M. polymorpha* gemmae and *A. thaliana* leaves (*P* values <0.0001, n${}_{M.\;\mathrm{polymorpha}}$= 15, n${}_{A.\;\mathrm{thaliana}}$= 23, Wilcoxon rank sum test). By contrast, new cell walls tested between 20 and 30 HAD appeared to be stiffer (Young’s modulus ratio >1) in *M. polymorpha* (n${}_{M.\;\mathrm{polymorpha}}$ = 16) until a stiffness similar to the surrounding walls is reached after the new wall has been bisected by a further cell division (*P* value <0.001, n${}_{M.\;\mathrm{polymorpha}}$ = 8, Wilcoxon rank sum test). In *Arabidopsis* leaves, new walls reach the same stiffness as the surrounding walls (Young’s modulus ratio ∼ 1) within 20 to 30 HAD (*P* values < 0.0001, n${}_{A.\;\mathrm{thaliana}}$ = 28, Wilcoxon rank sum test). (*D*) Schematic of the mechanical properties of walls used to compute Young’s modulus ratio. The blue wall is referred to as a new wall (numerator of the ratio); even after further divisions the new wall we refer to is still the original new wall (blue) and all measurements are of this wall. The purple walls are referred to as mother cell walls (denominator of the ratio). (*E*) A first *A. thaliana* true leaf is imaged every 24 h. The dividing cells are identified via color-coded markers across time points (< 6 h—yellow, 20 h>—red). (*F*) Atomic force microscopy of the imaged cells. Plot of the stiffness map and the contact point map of the clones tested at 8 DAS.

To assess the mechanical properties of the new cell walls, confocal microscopy was combined with local mechanical measurements of the cell wall’s mechanical properties using AFM. The confocal images provided information regarding cell wall age, while AFM provided information on the mechanical properties—i.e., stiffness. Time-lapse confocal microscopy was performed on gemmae starting from 18 HAG—when cell divisions started to occur, in agreement with the previous analysis—until 48 HAG. Imaging was focused on the notch region, identified as the area with the most dividing cells. New cell walls were identified in each image (Fig. 2*A*). Given that *M. polymorpha* gemmae are amenable to AFM measurements without prior dissection, cell wall properties were measured at both 24 and 48 HAG for the same cell lineages that had been previously imaged (Fig. 2*B*). By repeatedly measuring the same cell walls at 24 HAG and 48 HAG we were able to see that the new walls were initially soft then became stiffer compared to the surrounding parental walls. As a result, this preliminary qualitative examination revealed a temporary stiffening behavior in the newly developed cell walls in *M. polymorpha* gemmae. Further, by comparing the AFM height maps at 24 HAG and 48 HAG, we observed a change in the sample topology (contact point map). Immediately after cell division <6 HAD there was no change in the height of the outer periclinal epidermal wall; however, later 20 to 30 HAD there was a difference in the height of the wall. This observation suggests that the different properties of the soft and stiff walls are enough to generate this geometry change (Fig. 2*B*), suggesting that the anticlinal wall is restricting the periclinal wall from growing or inflating upward.

Note that the time of cell division is measured from the appearance of a new complete intersection of the cell (as observed for fluorescently labeled plasma membrane) during the confocal time lapse, identifying the moment when cytokinesis is completed. A 0.4-μm pixel size was used in AFM maps to obtain cell wall resolution during force measurements (*SI Appendix*, Fig. S4*A*). AFM measurements were performed within the limit of small deformations—i.e., maximum cell wall indentation 10% cell height. However, to check that newly formed walls were not damaged during the test, a ramp-up in force followed by a ramp-down was performed (*SI Appendix*, Fig. S4*B*). This test showed that cell wall mechanical properties at the beginning and end of the test were the same, suggesting that no detectable damage was induced to the cell wall by the testing conditions used here to generate the force data (see *Materials and Methods* for testing parameters).

Using the combination of confocal time course and AFM, new cell walls’ properties were quantified at different stages (Fig. 2*C*). We computed Young’s modulus ratio as the ratio between the average new wall Young’s modulus (blue) and the average mother cell Young’s modulus (purple) (Fig. 2*D*–see *Material and Methods* for details on the computation of the average Young’s modulus from AFM measurements). For further quantification of the mechanical properties, gemmae new walls were measured only once with AFM at the desired time after division to eliminate any possible effects of being repeatedly plasmolyzed. The samples were classified into three categories based on the age of the new cell wall (Hours After Division HAD): i) <6 HAD—i.e., hours since the appearance of the new wall in the confocal image; ii) 20 to 30 HAD; iii) 10 h after the mother cell has undergone a second round of divisions (a second round of division is considered when the first division wall is bisected at least once, see Fig. 2*D*). Very new walls (AFM measurements made less than 6 HAD) were roughly 70% softer than the parental walls (Fig. 2*C*). New walls that were 20 to 30 HAD, however, were 40% stiffer than the parental wall (Fig. 2*C*). This mechanical heterogeneity of the new cell walls was gradually lost after the cell underwent subsequent rounds of divisions and that new wall was bisected at least once by additional walls (Fig. 2*C*). The absolute values for the new wall Young’s modulus (numerator of the ratio) and the mother cell Young’s modulus (denominator of the ratio) are plotted in *SI Appendix*, Fig. S5*A*. Through the calculation of the height difference between the new wall and the adjacent anticlinal mother wall over time (*SI Appendix*, Fig. S5*C*), we observe a decrease in height difference between the new wall and the mother wall which correlated with their age—HAD (*SI Appendix*, Fig. S5*D*). To exclude that such stiffening behavior was an artifact of AFM tip size, force-maps were acquired with a smaller one (20 nm radius). These maps also showed that the walls of newly divided cells were stiffer than the original mother cell walls (*SI Appendix*, Fig. S4*C*). Finally, no correlation between stiffness and contact point height was identified within AFM maps, thus excluding the possibility that such cell wall stiffening measurements are the result of different cell wall heights (*SI Appendix*, Fig. S6*A*). Note that for this check, the contact point and Young’s modulus values for all the cell walls within the selected AFM maps were included, independently of their age.

## New Cell Walls Obtain Parental Stiffness by 24 Hours After Division in A. thaliana First True Leaves

To investigate the generality of our observation that new walls are stiffer than the parental walls, we repeated our experiment on the model species *A. thaliana*. Leaves were imaged every 12 h from 5.5 to 8 days after stratification (DAS) when the cells were dividing and elongating (Fig. 2*E* and *SI Appendix*, Fig. S7 *A* and *B*). As shown previously, cells tended to divide according to the shortest path across the cell (*SI Appendix*, Fig. S7 *C* and *D*) (17, 22). In contrast to *M. polymorpha* gemmae, first true leaves had to be dissected in order to perform AFM measurements, therefore, mechanical measurements of the clones were only performed at a one-time point (Fig. 2*F*). Comparing the cell wall stiffness to the age of the wall relative to the time of cell division showed that newly formed cell walls (<6 HAD) were softer than the surrounding cell walls of the mother cell. In contrast to *M. polymorpha*, *A. thaliana* walls 20 to 30 HAD had a similar stiffness to the average of the parental cell walls (Fig. 2*C*). Similarly to *M. polymorpha* gemmae, immediately after cell division <6 HAD there is no change in the height of the outer periclinal epidermal wall, however, later 20 to 30 HAD there is a difference in the height of the wall suggesting that the anticlinal wall is restricting the periclinal wall from growing or inflating upward (Fig. 2*F* and *SI Appendix*, Fig. S5*D*). Note that, while the final cell size and shape differ between mature *A. thaliana* leaves and *M. polymorpha* thalli (*SI Appendix*, Fig. S8*B*), the time-lapse imaging and AFM analysis were performed on young tissues with comparable cell shapes (*SI Appendix*, Fig. S8 *A* and *E*) and cell areas (*SI Appendix*, Fig. S8*D*, blue boxplots). Quantification of cell lobeyness in the young tissues (at the time of testing) of the two species shows that cells possess a similar polygonal geometry (*SI Appendix*, Fig. S8*E*, blue boxplots).

## Cell Wall Growth Inversely Correlates with the Measured Stiffness

**Fig. 3.**
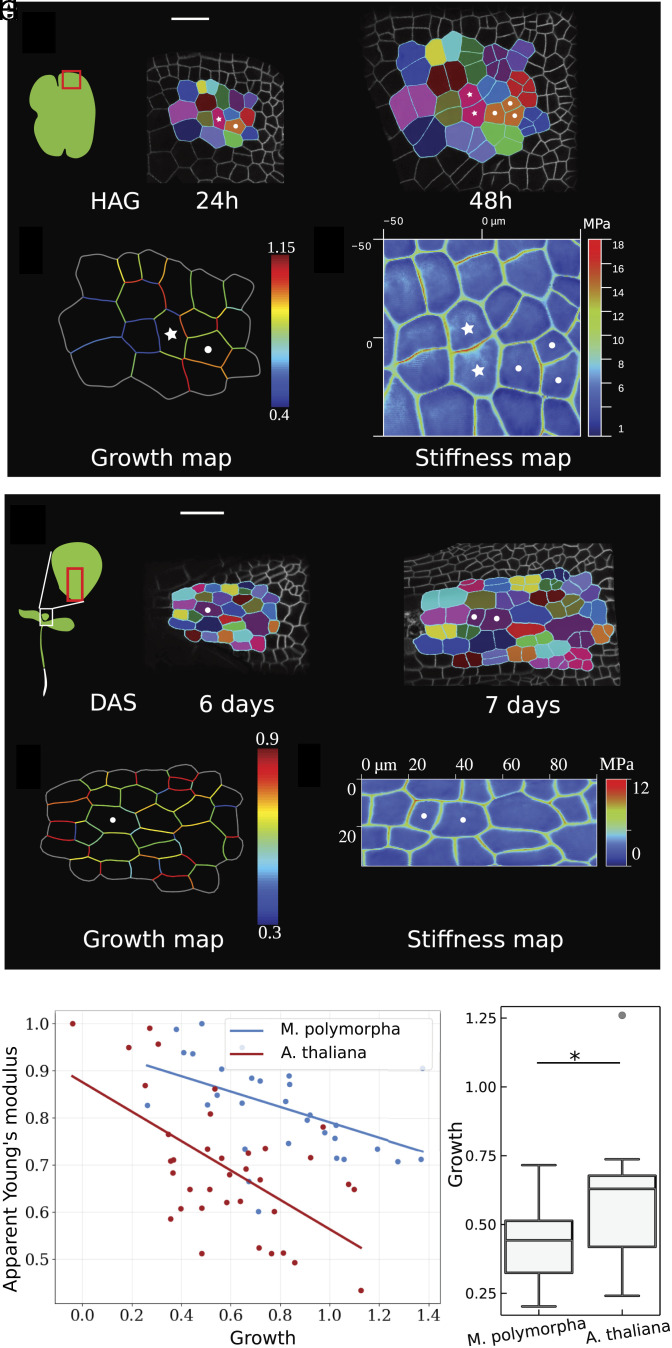
Cell wall stiffness correlates with growth in *M. polymorpha* gemmae and *A. thaliana* leaves. (*A*) Time course of a patch of cells in *M. polymorpha* gemma every 24 h, where the same clones are filled with the same color. (Scale bar, 50 μm.) (*B*) The growth map of cell walls is constructed using the ratio that compares the difference in length of cell walls between two time points to the length of the cell walls at the first time point; red cell walls are those that elongated the most, and blue cell walls are those that elongated the least. (*C*) The cell wall stiffness of the clones at 48 h is measured via atomic force microscopy, where red walls are the stiffer ones and blue walls are the softer ones. (*D*) Time course of a patch of cells in the dividing zone of the first true leaf of *A. thaliana* every 24 h, where the same clones are filled with the same color. (Scale bar, 50 μm.) (*E*) The growth map of cell walls is constructed using the ratio of change in cell length between the two time points and the length of the cell walls at the first time point; red cell walls are those that elongated the most, and blue cell walls are those that elongated the least. (*F*) The cell wall stiffness of the clones 7 DAS is measured via atomic force microscopy, where red walls are the stiffer ones and blue walls are the softer ones. (*G*) Inverse correlation between cell wall growth and apparent stiffness (i.e., stiffness is normalized with respect to the maximum value in the stiffness map). Cell walls from both *M. polymorpha* gemmae and *A. thaliana* true leaves show that cell walls elongating the most are the softest ones (see *SI Appendix* for additional samples and *SI Appendix*, Table S3 for fitted parameters). (*H*) Comparison of the strain of new walls in *M. polymorpha* (*n* = 15) and *A. thaliana* (*n* = 19). The growth of new walls around 24 HAD shows that new walls in *M. polymorpha* grow less than *A. thaliana* (*P* value = 0.028 < 0.05, Wilcoxon rank sum test).

While new cell walls undergo significant changes in mechanical properties, parental walls also show variability in their stiffness. To determine if such heterogeneity has a biological significance, we compared the stiffness measured with AFM for each wall with their growth. Patches of cells in *M. polymorpha* gemmae were followed with time-lapse microscopy for 24 h (Fig. 3*A*). The growth maps of individual cell walls were computed as the change in length between two time points over the original length—see *Materials and Methods* for code repository (Fig. 3*B*). The previously imaged cell walls were then identified under the AFM via preliminary coarse indentation resolution (*Material and Methods*), and their stiffness was measured with the previously optimized settings (provided in the *Materials and Methods*) (Fig. 3*C*). All walls which could be followed were included irrespective of age. The same study was then performed for the first true leaf of *A. thaliana* (Fig. 3*D*–*F*). We found that the individual walls of a cell (i.e., each edge of the polygonal cells) had different growth and there was a negative relation between the measured cell wall stiffness and growth during the observed time period (Fig. 3*G* and *SI Appendix*, Fig. S6*B*). We have reported above that new cell walls in *M. polymorpha* gemmae are stiffer than those in *A. thaliana* leaves—absolute value, *SI Appendix*, Fig. S5 *A* and *B*. Further, stiffer walls elongate less than soft walls. We also found experimentally that the new *M. polymorpha* walls elongate less than *A. thaliana* new walls 24 HAD providing further evidence of this correlation (Fig. 3*H*).

While comparisons of AFM maps collected using independently calibrated cantilevers must be carefully performed because of the intrinsic complexity of such technique, we consistently observed that the anticlinal cell walls in *M. polymorpha* gemmae are twice as stiff as those in *A. thaliana* leaves (e.g., Fig. 3 *C* and *F* and *SI Appendix*, Fig. S8*C*, quantification presented in *SI Appendix*, Fig. S5 *A* and *B*). This result was further confirmed by the fact that to indent *A. thaliana* leaves by ∼10% of their cell height a lower force was required.

## The Transient Stiffness of New Walls Impacts the Local Cell Geometry

**Fig. 4.**
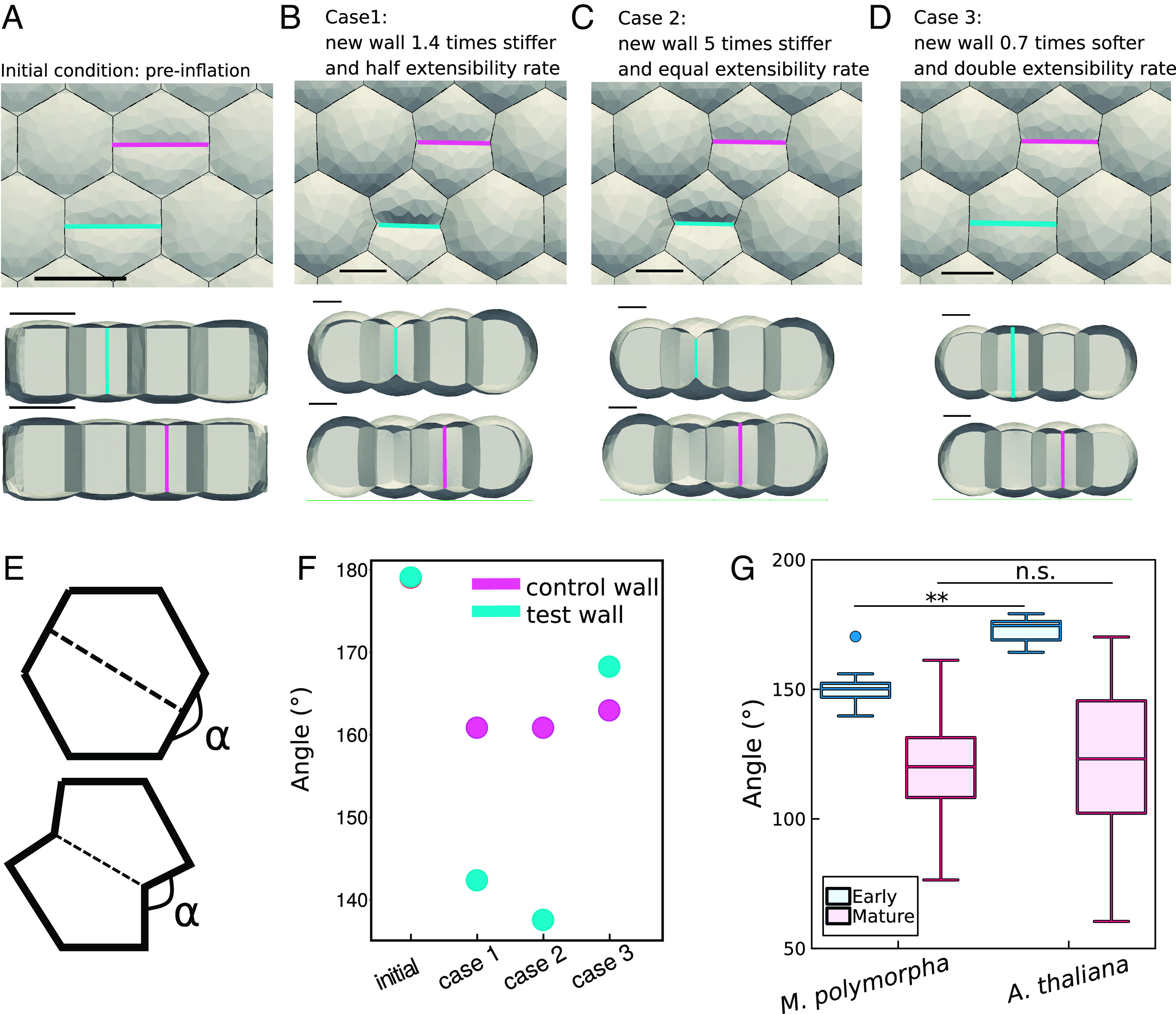
Cell wall stiffening of newly divided cells correlates with junction angle. (*A*–*D*) Simulations of the impact of a stiffer new wall on junction angle. A *Top* view and side view showing both the control wall, which has the same properties as the surrounding walls (magenta) and test wall, for which the parameters are altered (cyan). All Scale bars are 10 μm. (*A*) Initial conditions used as the starting point for the simulations, mesh was inflated with no growth and uniform parameters of a Young’s modulus = 100 MPa, turgor pressure = 0.2 MPa. All simulations had a turgor pressure of 0.2 MPa. During inflation, the control and test walls had the same parameters. (*B*) Case 1: the test wall (cyan) had a slower extensibility rate 0.1 h${}^{-}1$ (half) and a stiffer wall Young’s modulus of 140 MPa (40% increment) compared the other walls having a growth rate of 0.2 h${}^{-}1$ and a Young’s modulus of 100 MPa. (*C*) Case 2: the test wall (cyan) was much stiffer with a Young’s modulus of 500 MPa (five times the stiffness of the control wall). The control and surround walls had a Young’s modulus of 100 MPa. All walls had equal extensibility rate. (*D*) Case 3: the test wall (cyan) had a higher extensibility rate of 0.2 h${}^{-}1$ (double) and a Young’s modulus of 100 MPa (30% decrease) and the rest had an extensibility rate of 0.1 h${}^{-}1$ and a Young’s modulus of 140 MPa. (*E*) A diagram showing the pinch-in angle α which is used to compare the models. (*F*) Quantification of pinching-in of walls perpendicular to test and control walls in simulations *A*–*C* (see numerical values in *SI Appendix*, Table S5). (*G*) Quantification of the pinch-in angle 24 HAD in *A. thaliana* and *M. polymorpha* reveals the effect of a stiffer new cell wall on the wall perpendicular to cell division (for tissues at early stage: n${}_{M.\;\mathrm{polymorpha}}$ = 16, n${}_{A.\;\mathrm{thaliana}}$ = 18, *P* value < 0.001, for tissues at a mature stage: n${}_{M.\;\mathrm{polymorpha}}$= 216, n${}_{A.\;\mathrm{thaliana}}$ = 24, *P* value > 0.05 - n.s.; Wilcoxon rank sum test).

To further investigate the consequences of *M. polymorpha* having stiffer new cell walls, we simulated a tissue of pressurized 3D growing hexagonal cells using the Tissue modeling environment (41, 55–57); see *Material and Methods*. To simulate cell division, we added cross-walls to two of the inflated hexagons (Fig. 4*A*–*D*). One of the two dividing cells has a wall (magenta, *Top* dividing cell) with the same properties as the rest of the tissue, while the other (cyan, *Bottom* dividing cell) has different properties—i.e., different Young’s modulus (elastic stiffness) and/or extensibility rate (rate of irreversible deformation) (Fig. 4*A*–*D*). In our model, we characterize tissue growth by considering the elastic strain on the wall, which is inversely related to its Young’s modulus and captures the elastic behavior of plant cell walls. We account for the irreversible extension of that edge as a modification with a certain extensibility rate in the wall’s resting configuration. This modification is directly proportional to both the elastic strain and length of the wall (*Materials and Methods*). This approach was previously introduced to capture the main characteristics of the growth of plant cell walls (15, 57). Before simulating the growth behavior of the two daughter cells to assess the effect of the different properties of the new wall on the cell shape, all cells with homogeneous stiffness were preinflated [turgor 0.2 MPa, in agreement with previous studies in the literature (58, 59)] (Fig. 4*A*). Changes in wall properties may affect both the elastic stiffness (Young’s modulus as estimated in the AFM data) and the extensibility rate (related to both the ability of the wall to creep, and addition of new material). To capture this, we first simulated the scenario resembling the experimental observations (Case 1). The new wall (the test case wall, cyan wall) is modeled 40% stiffer and, to mimic the resulting growth in planta, the extensibility is set to half that of the walls of the mother cell (Case 1, see parameters in *SI Appendix*, Table S4). We observed the impact of the altered cell wall properties on the shape of the mother cell, as defined by the amount of deformation of the originally straight mother cell wall referred to as the pinching-in angle (Fig. 4*E*). The pinching-in angle in the cell shape is measured in the model when the total cell area of the two daughter cells is twice that of the original mother cell—consistent with the experimental observations of the cells after 24 h (*SI Appendix*, Fig. S9*A*). Both the control and the test wall caused a pinching-in of the mother cell walls due to the mechanical properties of the walls restricting expansion, consistent with previous 2D models. The restricted expansion of the new wall restricts the mother cell wall such that its shape is altered. There is negligible pinching-in in the initial inflation step where only the elastic strain is considered (60). However, the deformation of the mother cell wall is greater in the test case 142${}^{{}^{\circ}}$ compared to 160.5${}^{{}^{\circ}}$ of the control case (Fig. 4 *B* and *F*). To assess if the difference in extensibility rate (thus growth) between the new wall and the surrounding one was necessary to explain the pinch-in angle, only Young’s modulus of the new wall is changed in the following simulation (Case 2, see parameters in *SI Appendix*, Table S4). In order to achieve a pinch-in angle comparable to that when growth was also altered, the new wall must be 5 times stiffer than the surrounding walls (Fig. 4*C* and *F*). In both Case 1 and Case 2 we see an out-of-plane deformation caused by the stiffer wall restricting the periclinal wall (Fig. 4 *B* and *C*), which was also observed in the AFM data (Fig. 2*B*). To further confirm the interplay of stiffening and extensibility differences on cell shape after division, we show that the effect of a softer wall with double the extensibility rate with respect to the surrounding ones is neglectable on the final cell shape (Case 3, see parameters in *SI Appendix*, Table S4 and Fig. 4 *D* and *F*). It is important to note that in this case, both the pinch-in angle (Fig. 4*F*) and the out-of-plane deformation of the periclinal wall are absent (Fig. 4*D*, *Bottom*), and this can be explained by the surrounding walls bearing the load.

In conclusion, the computational study provides evidence that the presence of a stiff new cell wall accelerates the convergence of the junction angle toward a value of 120${}^{{}^{\circ}}$ by deforming the growing mother cell wall, which is the characteristic angle of a cell junction in mature tissue. We compared the pinching-in angle in *M. polymorpha* and *A. thaliana* in tissues at a mature stage to see if it was impacted by the difference in new wall stiffness and growth. In accordance with previous studies (61), we found that the average junction angle achieved in both species was 120 (Fig. 4*G*). However, in the first 24 h following cell division, the junction angle in *M. polymorpha* was smaller than in *A. thaliana* suggesting that the stiffer new wall created a faster convergence toward the junction angle of 120${}^{{}^{\circ}}$. The model also suggests that the observed cell shape after cell division is the result of both a stiffer new wall and a difference in wall extensibility. Although the modeled extensibility rate and observed growth rate do not align perfectly, the model demonstrates that solely modifying the elastic properties of the wall would require a very high (five-fold) difference in mechanical stiffness to achieve a pinching-in angle comparable to the experimental observation. Hence, this evidence suggests that the altered wall stiffness acts by also influencing cell wall growth, resulting in the accelerated pinching-in observed in *M. polymorpha*, in accordance with experimental findings.

## Discussion

This work addresses the spatial and temporal modulation of the mechanical properties of cell walls during cell division and cell expansion by combining time-lapse imaging with local mechanical measurements via atomic force microscopy. The study makes use of two model systems: *M. polymorpha* gemmae and *A. thaliana* leaves. The *A. thaliana* leaf is a well-established model system to study plant morphogenesis and it has been widely shown that cells divide along the shortest path. Here, we have shown that the new cell walls in *M. polymorpha* gemmae align more with cellular geometry at early time of development (<36 HAG), while microtubule orientation becomes a better predictor at later stages of development (>36 HAG). Assuming that microtubules align with mechanical stress, this result suggests that geometrical clues are overruled by tissue level stresses 36 HAG. It has previously been shown computationally that the deposition of new materials along the direction of maximum stress during cell division allows the plant to locally redistribute the stress, thus locally unloading the old walls (28). Therefore, the location of new cell walls may be determined by *M. polymorpha* gemmae to reduce the stresses within the tissue. Further, the additional cell wall stiffness may reflect the growth environment of *M. polymorpha* where the cells are exposed to high biotic and abiotic stress.

Heterogeneous growth has been reported previously and it has been correlated with heterogeneous cell wall stiffness at the tissue level (62–64). Heterogeneous growth has also been observed within pavement cells (65). Building on these previous studies, we were able to directly correlate the stiffness heterogeneity of mature cell walls with growth at the individual cell wall level. Further, in our work we demonstrated that cell division can result in a heterogeneous pattern of cell wall stiffness, thus further affecting heterogeneous tissue growth. By combining time-lapse imaging and AFM, we have been able to show that the newly placed walls’ mechanical properties change over time. The lower Young’s modulus of newly placed cell walls that we observed in *A. thaliana* leaves and *M. polymorpha* gemmae is consistent with previous studies in *A. thaliana*. This apparent softer behavior can be associated with the time needed for the deposition and integration of new wall components (66, 67). Our data demonstrate a subsequent age-dependent transient stiffening of the new cell wall that has not been previously characterized. In *A. thaliana* leaves, new walls reach parental stiffness in 24 HAD. By contrast, in *M. polymorpha* gemmae, new walls reach 1.4 times parental wall stiffness 24 HAD. We showed in *M. polymorpha* the relative quantities of unesterified homogalacturonan was higher in the older nondividing tissue, pointing to differences in cell wall composition and architecture in young versus old cell walls. It is not clear what proportion of total pectin the former contributes although the degree of methylesterification can be linked to mechanical outputs, and the changes we observe in other cell wall components suggest marked remodeling of the wall as a tissue ages. We propose such difference may underlie the mechanical and subsequent growth differences in *M. polymorpha* gemmae compared to *A. thaliana* leaves. Further work in this area is required to quantify exactly and identify these differences at both the tissue and the individual cell wall levels, and to correlate them with cell wall age.

Models of cell division have often postulated that new walls should have reduced growth in order for expanding cells to obtain realistic hexagonal geometries and topologies (26, 68). Our data from *M. polymorpha* gemmae, which have largely polygonal-shaped cells, provide experimental support to these models. Through the use of 3D mechanical models, we were able to demonstrate that the experimentally observed higher cell wall stiffness and reduced growth rate of new walls result in the parental wall pinching-in more quickly and converging more quickly toward 120${}^{{}^{\circ}}$. *A. thaliana* leaf cells did not show the same amount of stiffening and also took longer to generate 120${}^{{}^{\circ}}$ angles at junctions. While the work highlights how new cell walls affect the cell shape locally, further work is needed to quantify how this age-dependent heterogeneity (both stiffness and growth) affects morphogenesis at the tissue level.

Finally, mechanical stress has also been shown to be a regulator of plant development, and differential wall/membrane tension could influence cellular mechanical stress and play a role in the polarity of key developmental regulators such as PIN1 (69, 70). Models have often speculated that the properties of newer and older walls may be different and that this could have a role in cell polarity (17, 71, 72). Our data support this possibility and open the field to further investigate this hypothesis.

## Materials and Methods

Experimental data are available at the following Zenodo repository (73).

### Plant Lines and Growth Conditions.

The lines used in this study are described in *SI Appendix*, Table S1. All *M. polymorpha* gemmae were cultivated from gemmae under axenic conditions. All gemmae were grown on one-half-strength Gamborg B5 Basal (Duchefa Biochemie Cat.No G0209) media (pH 5.7) without B5 vitamins with 1% sucrose under continuous light (70 μE m${}^{-2}$ s${}^{-1}$) at 22 ${}^{{}^{\circ}}$C. Gemmae for experiments were taken from 3- to 4-wk-old plants, and they were grown on one-half-strength Gamborg B5 Basal media (as above) without sucrose under the same conditions.

*A. thaliana* seeds were surface sterilized and sown on 1/2 MS. Seeds were stratified for 2 d at 4${}^{{}^{\circ}}$, then transferred to long-day 16 h light 8 h dark, 20 ${}^{{}^{\circ}}$C. Seedlings were grown vertically.

### Cloning and M. polymorpha Transformation.

*M. polymorpha* TAK1 (“GA” stock) thalli were cotransformed with the MpEF1::GFP-MpTUB1 [Hygromycin resistance (74)] and MpEF1a::myr-mScarlet (Chlorsulfuron resistance–pMpGWB303) constructs. Myristolated-mScarlet was cloned using a multistep PCR approach with overlapping primers to generate the myristolation signal sequence on the N terminus of mScarlet. The final amplicon was flanked by universal attB sites that enabled BP recombination (Gateway BP Clonase II, Invitrogen) into pDONR221 following the manufacturer’s instructions. A sequence verified myr-mScarlet entry clone was subsequently used to generate the MpEF1a:myr-mScarlet expression construct by LR recombination into pMpGWB303 (Addgene no. 68631) (75) using LR Clonase II (Invitrogen) following the manufacturer’s instructions. The resulting construct was transformed into Agrobacterium tumefaciens GV3101 (pMP90) by electroporation. Primers described in *SI Appendix*, Table S2. *M. polymorpha* transformation were performed using the Agrobacterium-mediated thallus regeneration method (76) in the TAK1 background. The dual-labeled reporter line containing MpEF1a::myr-mScarlet (this study) and MpEF1a::GFP-MpTUB1 (74) reporter line was generated by cotransformations that were selected on solid 1/2 strength MS-B5 media supplemented with cefotaxime (125 μg/mL), hygromycin B (20 μg/mL), or chlorsulfuron (1 μM). Stable transgenic liverworts were obtained by propagating mScarlet and GFP fluorescent gemmae from T1 thalli. Experiments were performed in the second gemmae (G2) generation or in subsequent generations.

### Atomic Force Microscopy Experiments.

Gemmae were taken from 3- to 4-wk-old plants and grown on one-half-strength Gamborg B5 Basal media for 1 d. One-day-old *M. polymorpha* gemmae were immobilized on one-half-strength Gamborg B5 Basal media (Duchefa Biochemie Cat.No G0209) with 2% agarose. *A. thaliana* leaves were immobilized on 2% agarose gel. Both *M. polymorpha* gemmae and *A. thaliana* leaves were immobilized in 35- × 10-mm Polystyrene petri dishes (Merck CLS430165). The cantilever used was “Nanotools” biosphere B150-NCH with spherical tip of radius 150 nm (0.3 μm diameter) and spring constant of 40 N/m. In order to extract the mechanical properties of the walls, all samples were plasmolyzed in 10% (0.55 M) sorbitol solution for 10 min before starting the experiment. The speed of indentation was 100 μm/s, and the maximum indentation force was, respectively, ∼700 nN for *M. polymorpha* gemmae and ∼400 nN for *A. thaliana* leaf to achieve a maximum indentation of 0.2 to 0.5 μm (<1% deformation). The AFM cantilever was used to indent the samples over an area varying between 50 × 50 to 100 × 100 μm with a pixel size of 0.4 μm. Each force-indentation curve was fitted with a Hertzian indentation model to extract an apparent Young’s modulus. The apparent Young’s modulus was calculated using the JPK Data Processing software (software version 6.4). Only the approach curve was used for the analysis. A Poisson ratio of 0.5 was assumed for the material. Analysis of the AFM force maps and extraction of individual walls’ property were performed using the open-source software Gwyddion (77). Note that to identify the same cells imaged with the confocal during the time course, preliminary indentation maps with a pixel size of 2 μm and indentation force of 400 nN (for *M. polymorpha* gemmae), 200 nN (for *A. thaliana* leaves), were collected. The cantilever was then moved at the location of the desired cells.

#### Computation of the average Young’s modulus.

Young’s modulus ratio is computed as the ratio between the average Young’s modulus of the new cell wall and the average Young’s modulus of the surrounding walls of the mother cell (see schematic in Fig. 2*D*). To extract Young’s modulus of the new wall, first a mask is first defined in Gwyddion using the line tool (2 pixels thick). Next, this mask is applied to Young’s modulus map using the “Arithmetic operation on data” function. This is done by multiplying Young’s modulus map with the created mask. As a result, only Young’s modulus values corresponding to the pixels selected in the mask are extracted. The average Young’s modulus for the new cell wall is finally computed as the average value of all extracted pixels. The same procedure is performed to extract the average Young’s modulus for the mother cell walls. In this case, the surrounding walls of the mother cell are selected within the mask (Fig. 2*D*). The same procedure is also performed to extract the average Young’s modulus for each individual wall to study the correlation between growth and stiffness.

#### Computation of the height difference.

The height difference is computed as the difference between the average contact height of the new cell wall and the average contact height of the surrounding walls of the mother cell (see schematic in Fig. 2*D*). To extract the contact height of the new wall, a mask is first defined in Gwyddion using the line tool (2 pixels thick) on Young’s modulus map since the walls are more visible and defined. Next, this mask is applied to the contact point map using the “Arithmetic operation on data” function. As a result, only the contact point values corresponding to the pixels selected in the mask are extracted. The average contact height for the new cell wall is finally computed as the average value of all extracted pixels. The same procedure is performed to extract the average contact height for the mother cell walls. In this case, the surrounding walls of the mother cell are selected within the mask (Fig. 2*D*).

### Confocal Imaging.

Timelapse images of both *M. polymorpha* gemmae and *A. thaliana* leaves were acquired using the confocal microscope Leica SP8. myrScarlet/GFP-TUB1 was excited with a 488-nm laser at 0.7% and a 552-nm laser at 0.3%, emissions were collected, respectively, at 500 to 520 and 584 to 610. Images for plasma-membrane time-series were acquired of format 1,024 × 1,024 pixel, with a speed of 400, bidirectional ON. MBD-GFP was excited with 488-nm laser at 0.7%, emission was collected at 500 to 520. Td-tomato was excited with 552-nm laser at 1.8%, emissions were collected at 570 to 590. Images of microtubules were acquired of format 1,600 × 1,600, speed 400, line average of 2, bidirectional ON, pinhole of 0.95, z-step of 0.4 to 0.5 μm. *M. polymorpha* gemmae were imaged in water within the Petri dish in which they had been growing. Prior to imaging of *A. thaliana* leaves, *A. thaliana* seedlings were transferred in a small Petri dish and immobilized with 1/2 MS media. Leaves were also submerged in water for imaging.

Imaging of L1 and L2 microtubules in *M. polymorpha* was performed in two steps. First, the L1 layer was imaged using the settings reported above. Second, the L2 layer was imaged by changing the depth of focus and by increasing the laser power to a maximum of 4%. The other parameters remained unchanged.

### Image Analysis.

#### Segmentation.

Segmentation of time-series images was performed using MorphoGraphX (39, 40). The external shape of the object was extracted using the “Edge detect” process. The extracted surface is meshed using the process “Marching Cubes Surface”—with 5-μm cube size. After subdividing twice and smoothing the mesh, the signal was projected onto the mesh. Cell centers were identified using the autosegmentation process, and errors in the segmentation were subsequently manually corrected. Similarly, quantification of the microtubule orientation was obtained after the segmentation still using MorphoGraphX, using the adapted version of the ImageJ plug-in FibrilTool (44). Prior to quantification, microtubule images were preprocessed using the deconvolution module of Huygens Essential—default parameters were used.

#### Division rule.

For the computation of the shortest path in cell division ad hoc scripts implementing the approximation of new cell walls to circumference arches (22) were developed in Julia. The geometry of the dividing cell is obtained from the MorphoGraphX mesh prior to division. A plane is created by fitting it to the vertices of the cell, and the geometry is then rotated to align with a plane parallel to the XY plane. The same process is applied to the same cell after division, ensuring both geometries are registered (alignment procedure). The centroid of the geometry before division is determined. All arches connecting two points on two nonconsecutive walls passing through the centroid are identified. For all the arches within 2% of the shortest one, the angle difference with the actual plane of division is calculated. This choice was made to avoid that in case of two competing shortest paths (e.g., in a square), the one with the highest angle was chosen (22). The 2% of the wall length is well within the resolution of the segmentation. The division walls are computed as arches; therefore, the orientation of the path is computed as the tangent to the arc at the center of the cell. The arch with the lowest angle difference is selected as the shortest path. Maximum projection of cortical microtubule signal on the extracted cell shapes shows the main microtubule orientation, while a comparison of the cell shapes between the two different time points allows the calculation of the maximum growth direction. Both analyses are performed using MorphoGraphX (via respectively the FibrilTool plug-in and the Principal Growth Direction process). When the angle between the actual plane of division and the prediction orientation is below 15${}^{{}^{\circ}}$, for both shortest path and microtubule orientation, the two predictions are considered in agreement.

#### Cell wall growth.

Quantification of the individual cell wall growth was performed by comparing the length of each cell wall between two different time points (t, t+δt): (L${}^{t+\delta t}$ - L${}^{t}$)/L${}^{t}$. This was achieved with ad hoc scripts developed in Julia. The script extracts the length of the walls at both time points from the segmented images, calculates the difference in length between the cell walls at the two time points, and divides the length difference obtained in the previous step by the length at the first time point. This yields the relative growth of the cell walls over the time interval between the two time points.

#### Angle calculation.

##### For new walls.

Angle calculation was performed using the “Angle tool” in Fiji. New cell walls 24 h old were first identified in the time-lapse analyses. Then, three points required for angle calculation were manually selected: 1) the first extreme of the old wall perpendicular to the new wall; 2) the intersection between the new wall and old wall; and 3) the second extreme of the old wall perpendicular to the new wall.

##### For mature walls.

Outlines were extracted using MorphoGraphX. Cell junctions were identified, we then calculated the vectors pointing outward from each junction between the junction center and average of the points 5 μm along the cell perimeter. The angles between these vectors were then calculated.

All scripts for the data analysis are publicly available and documented at the following GitHub repository (78).

### Statistical Analysis.

Statistical analysis was performed in Julia, using a two-sided Wilcoxon rank sum test that does not assume normality of the data. To determine whether a single dataset was significantly different from zero, a Wilcoxon signed rank test in Julia was used (79). To determine if a given sample of data is drawn from a given probability distribution, the Anderson–Darling test in Julia was used (79). Datasets with *P* values < 0.0001 were deemed to be highly significantly different and are denoted by three asterisks (***). Datasets with *P* value < 0.01 were deemed to be significantly different and are denoted by a double asterisk (**). Datasets with *P* value < 0.05 were deemed to be different and are denoted by a single asterisk (*). Changes with *P* value > 0.05 were considered nonsignificant (n.s.). For all box plots, the edges of the box represent the 25th and 75th percentiles of the data, the middle line marks the median. Line regression between cell wall Young’s modulus and growth (Fig. 2*G* and *SI Appendix*, Fig. S6*B*) was performed in Julia, using the GLM library (80). The results were confirmed twice by computing the regression with both LsqFit (81) and LinearRegression libraries (82).

### Immunostaining Marchantia Gemmae.

The immunostaining protocol and image analysis procedure of M. polymorpha gemmae are provided in *SI Appendix*, *Text*.

### CryoSEM Imaging.

CryoSEM was carried out as described in ref. 83 with the following modifications: A. thaliana leaf surface imaging was carried out using the Backscattered Electron detector and a gun voltage of 25 kV and Optibeam set to Depth Mode.

### Modeling.

Details of how the model was built are provided in *SI Appendix*, *Text*. A list of model parameter values can be found in *SI Appendix*, Table S4.

## Supplementary Material

Appendix 01 (PDF)

## Data Availability

All data; codes data have been deposited in Zenodo; GitHub (https://doi.org/10.5281/zenodo.7685356 and https://github.com/alebonfanti/plant-cell-division-growth) (73, 78).
