# Supplementary material for: Stiffness transitions in new walls post-cell division differ between Marchantia polymorpha gemmae and Arabidopsis thaliana leaves
Source: Proc Natl Acad Sci U S A. 2023 Oct 2;120(41):e2302985120. doi: 10.1073/pnas.2302985120 (PMC10576037; doi:10.1073/pnas.2302985120)
Supplement: Supplementary file 1 — Appendix 01 (PDF) [file pnas.2302985120.sapp.pdf]

Supporting Information for

Stiffness transitions in new walls post-cell division differ between *Marchantia polymorpha* gemmae and *Arabidopsis thaliana* leaves

Alessandra Bonfanti, Euan Thomas Smithers, Matthieu Bourdon, Alex Guyon, Philip Carella, Ross Carter, Raymond Wightman, Sebastian Schornack, Henrik Jönsson, Sarah Robinson

To whom correspondence should be addressed. E-mail: sarah.robinson@slcu.cam.ac.uk

This PDF file includes:

Supporting text
Figs. S1 to S9
Tables S1 to S5
SI References

13 ## Supporting Information Text

Table S1. Plants used in this study

Name	Species	Reference/Source
TAK1	*M. polymorpha*	Wild type male from Giulia Arsuffi (Kyoto International Marchantia Training Course, December 2016)
myrScarlet/GFP-TUB1 (mSgT)	*M. polymorpha*	This study
35S-MBD-GFP	*A. thaliana*	(1)
td-tomato	*A. thaliana*	(2)

Table S2. Primers used in this study

Primer	Sequence
attB1_uni	GGGGACAAGTTTGTACAAAAAAGCAGGCT
attB2_uni	GGGGACCACTTTGTACAAGAAAGCTGGGT
myr-attB1-F2	AAAAAGCAGGCTATGGGAGGATGCTTCTCTAAGAAGGTGA
myrScarlet-F1	AGAAAGCTGGGTCTACTTGTACAGCTCGTCCATGCCG

Table S3. Fitting coefficients of the linear regression (a and b) and R^2 value for the fitted line.

Sample type	a	b	R^2
M. polymorpha gemma AFM time course S01	-0.22	0.76	0.17
M. polymorpha gemma AFM time course S02	0.29	0.79	0.19
M. polymorpha gemma S01	-0.07	0.81	0.03
M. polymorpha gemma S02	-0.16	0.95	0.22
M. polymorpha gemma S03	-0.15	0.86	0.42
A. thaliana leaf S01	-0.31	0.88	0.35
A. thaliana leaf S02	-0.25	0.94	0.23
A. thaliana leaf S03	-0.09	0.92	0.22

Table S4. Computational model parameters. Properties of the new wall (stiffness and growth rate) and properties of the surrounding walls (stiffness and growth rate).

Simulation	New cell wall		Old cell wall	
	Stiffness	Extensibility rate	Stiffness	Extensibility rate
Control	100 MPa	0.2 hr^{-1}	100 MPa	0.2 hr^{-1}
Case 1	140 MPa	0.1 hr^{-1}	100 MPa	0.2 hr^{-1}
Case 2	500 MPa	0.2 hr^{-1}	100 MPa	0.2 hr^{-1}
Case 3	100 MPa	0.2 hr^{-1}	140 MPa	0.1 hr^{-1}

Table S5. Computational model results. The pinch-in angles, the new division walls' lengths and areas from the inflation study of a patch of hexagonal cells showing the consequence of different wall properties compared to a control after a hexagon has been divided in two. All results were taken when the cells of interest grow and exceed the same set area. Initial conditions used as the starting point for the simulations, mesh was inflated with no growth and uniform parameters of a Young's modulus= 100 MPa, turgor pressure= 0.2 MPa that will be the value used for all the simulations.

Simulation	Angle (°)	Area (μm^2)	New wall length (μm)	Change in new wall length (%)	Control angle (°)	Control area (μm^2)	Control wall length (μm)	Change in control wall length (%)	Timesteps
Initial conditions	178.8	82.4	10.7		178.7	82.4	10.7		0
Case 1	142.0	252.0	13.5	25.5	160.5	270.3	15.7	46.5	38
Case 2	137.2	248.0	12.8	19.4	160.5	269.0	15.6	45.9	105
Case 3	167.9	250.0	17.0	58.0	162.6	224.9	14.9	39.3	130

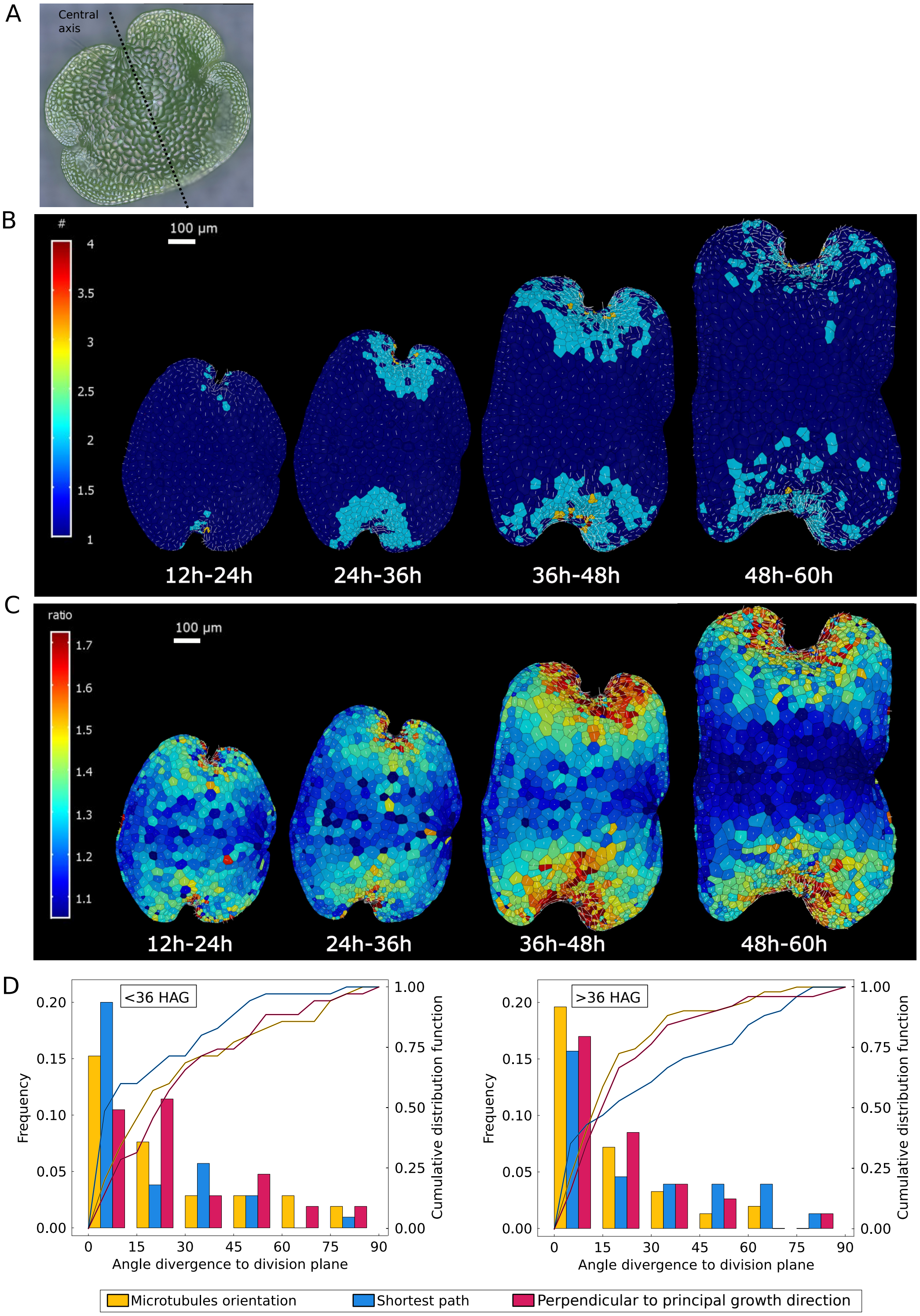

Fig. S1. *M. polymorpha* shape, growth and cell division patterns are symmetric with respect to the central axis. (A) Brightfield image taken with the digital microscope Keyence VHX-7000 of a 1 day old gemma. The plant possesses a symmetrical shape with respect to the central axis. (B) Cell division patterns of a full gemma for 60 hours. Colour maps show the number of divisions every 12 hours, scale bar 100 μm. (C) Growth map of a full gemma for 60 hours every 12 hours, where the red cells are those growing the most and the blue cells are those with a slower growth rate, scale bar 100 μm. (D) Quantification of angles between the actual plane of division and: (i) MT main orientation (yellow distribution), (ii) shortest path (blue distribution), and (iii) the Principle Growth Direction (pink distribution) for all the cell divisions occurred before 36 HAG (number of events = 35) and all divisions occurred after 36 HAG (number of events = 53). The yellow, blue and pink solid lines represent respectively the cumulative distribution for the three predictions–MT orientation, shortest path and direction perpendicular to Principal Growth Direction. A comparison of the cumulative distributions for the MT orientation and the shortest path shows that cell division in *M. polymorpha* better follows the shortest path for the events occurring before 36 HAG (Anderson-Darling test p-value = 0.0061), while for events 36 HAG the MT orientation is a better predictor (Anderson-Darling test p-value = 0.0128), which is a proxy for tension direction. The direction perpendicular to PGD shows good alignment with microtubule orientation and it thus is also a good predictor for division orientation after 36HAG (p-value between perpendicular to PGD and microtubule orientation: <36H = 0.89 n.s, >36HAG= 0.84 n.s.- Anderson-Darling test).

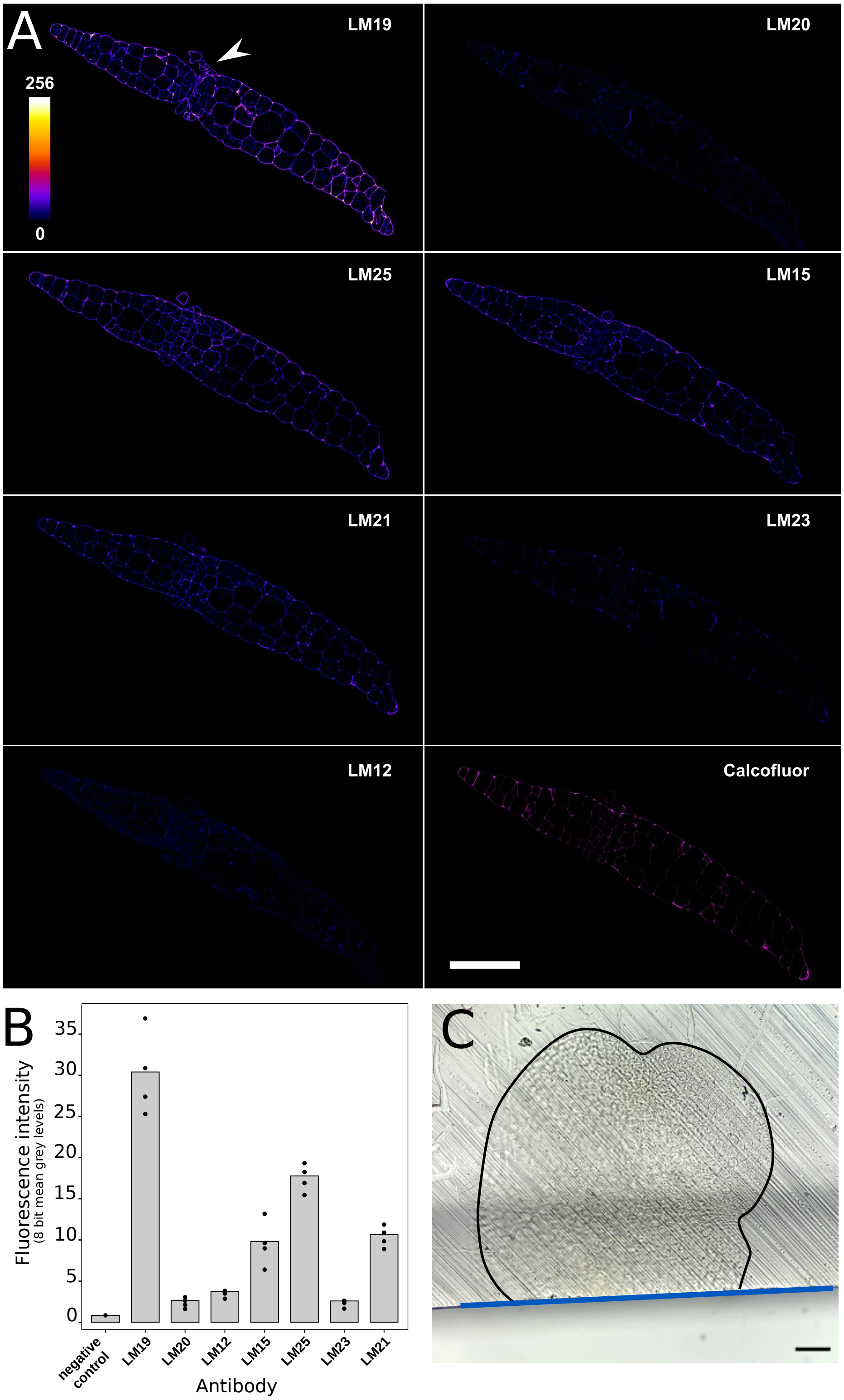

Fig. S2. (A) Comparison of cell wall epitopes immunostaining signal intensity. Visual comparison of immunostaining signals from several cell wall epitopes on 48h old gemmae cross-sections through the meristematic area. Images are represented in Fire LUT with a 8 bit colour coding of original grey levels (256 levels) represented on the figure top left. An example of cell wall counterstaining (calcofluor) is also represented. Arrowhead indicates the position of the meristematic area. Scale bar: 100 μm. (B) Immunolabeling quantification in early gemmae (2 days old) shows that LM20 fails to detect methyl-esterified pectin in *M. polymorpha* gemmae. (C) Bright field image of the sectioned gemma for immunostaining where the blue line indicates the position of the section and the black contour is the gemma edge (scale bar 100 μm).

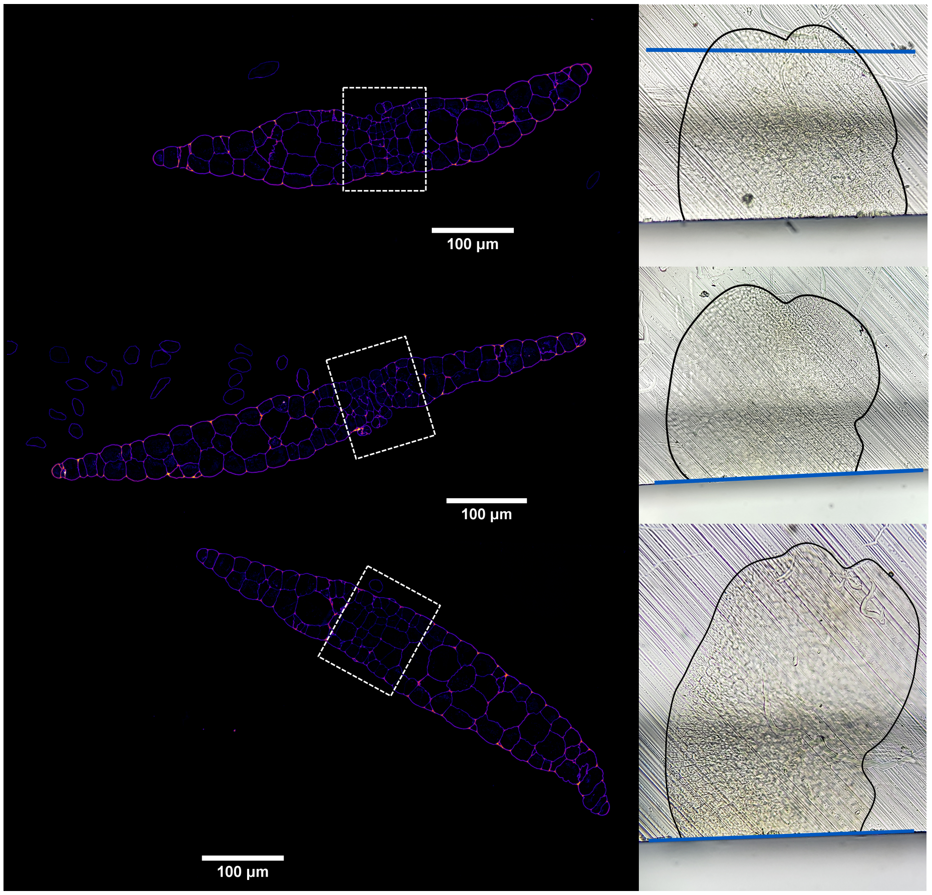

Fig. S3. LM19 immunostaining cross sections on 48 HAG *M. polymorpha* gemmae. Images are represented in Fire LUT with a 8 bit colour coding of original grey levels (256 levels). A dashed-white box has been placed to indicatively highlight the dividing zone. Bright field images of the sectioned gemma for immunostaining, where the black curves indicate the contour of the gemmae and the blue thick lines indicate the location of the sections for each sample.

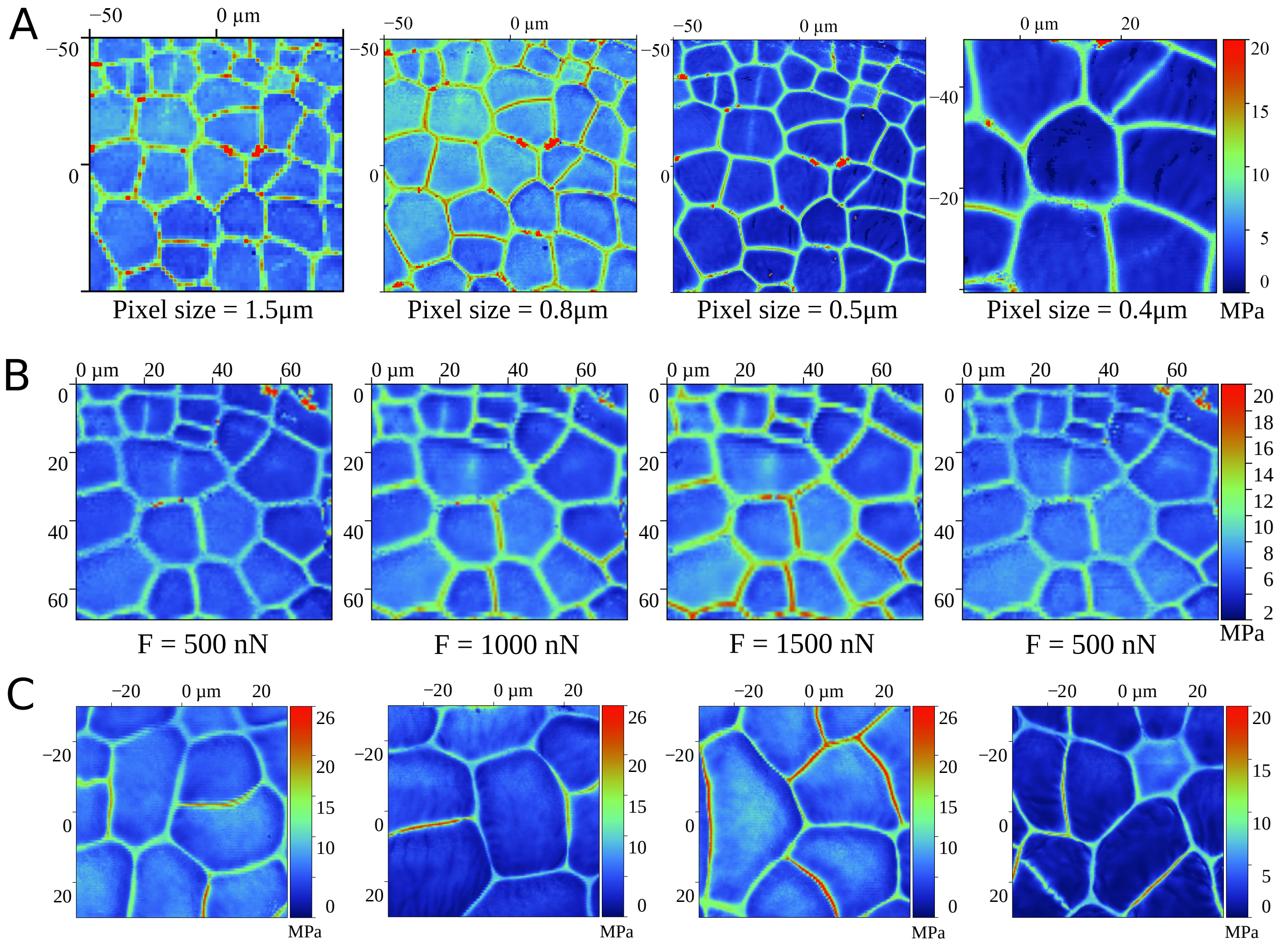

Fig. S4. Atomic Force Microscopy testing parameters. (A) AFM testing of a patch of cells in *M. polymorpha* gemmae with increasing number of points in the x and y direction - referred to as pixels–to be able to identify cell walls. The selected pixel size is 0.4 μm. (B) AFM testing of a patch of cells with increasing force followed by a final test with the original force. This shows that cell walls–even the new ones–are not damaged during the indentation at 10% deformation–corresponding to 600 nN. (C) Testing of *M. polymorpha* gemmae cells using a cantilever with tip radius of 20 nm.

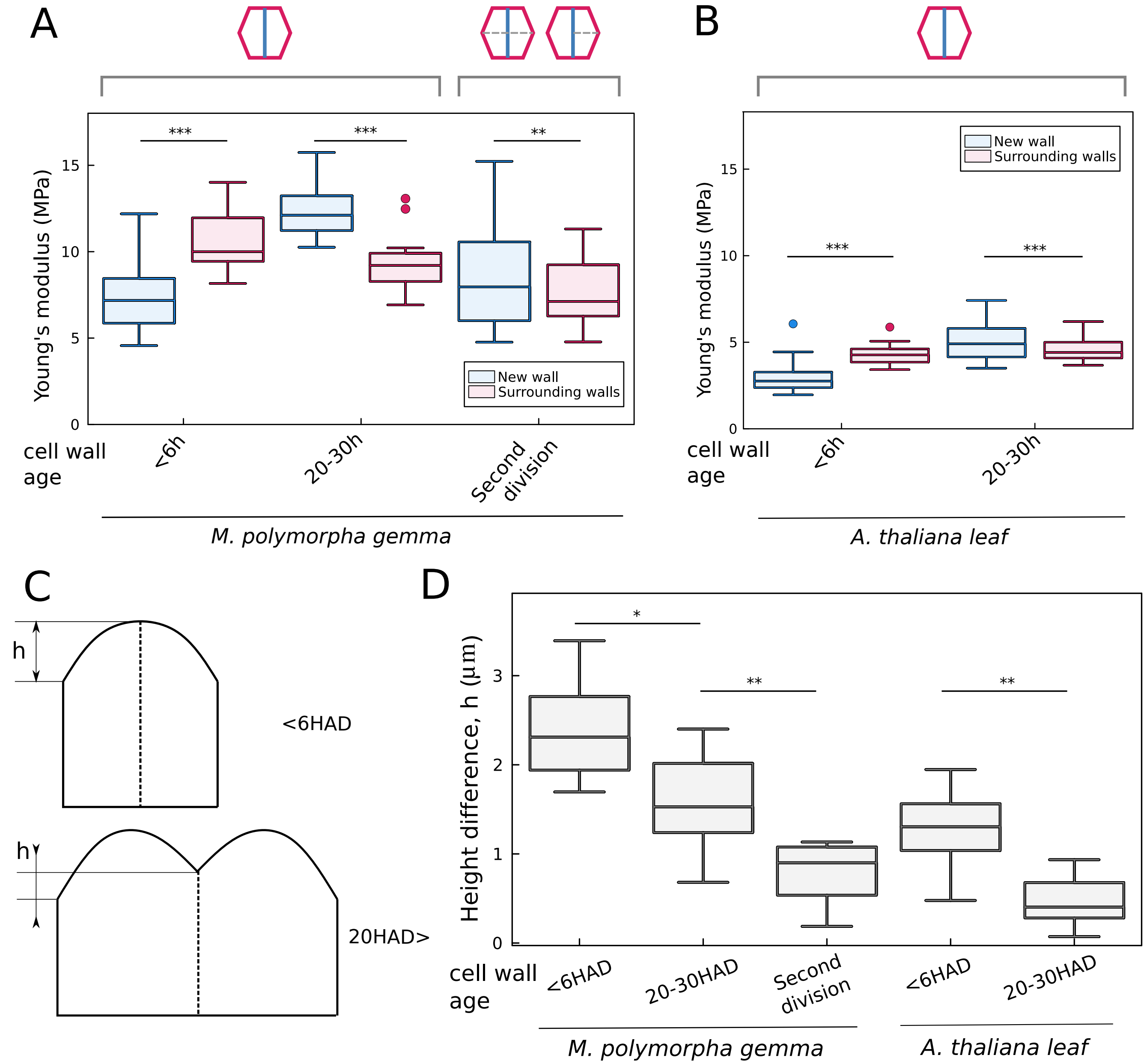

Fig. S5. Plotting of the average apparent Young's modulus of the new cell wall (blue) and the average apparent Young's modulus of the surrounding walls of the mother cell, against new cell wall age (Hours After Division). A schematic on top of each boxplot highlights which walls have been considered in the apparent Young's modulus calculation for the new wall and the mother cell walls. (A) The first plot refers to *M. polymorpha*, (B) the second plot refers to *A. thaliana*. In summary, the plots show that the stiffness of newly formed walls (<6 HAD) is lower than the surrounding walls of the mother cell. This is true for both *M. polymorpha* gemmae and *A. thaliana* leaves (p-values <0.0001, $n_{M.\ polymorpha}$= 15, $n_{A.\ thaliana}$= 23). By contrast, new cell walls tested 20-30 HAD appeared to be stiffer in *M. polymorpha* (p-value <0.0001, $n_{M.\ polymorpha}$= 16) until a stiffness similar to the surrounding walls is reached when a full second round of division is completed (p-value <0.001, $n_{M.\ polymorpha}$= 8). In *A. thaliana* leaves, new walls reach the same stiffness as the surrounding walls within 20-30 HAD (p-values <0.0001, $n_{A.\ thaliana}$= 28). All p-values are computed as Wilcoxon Rank Sum Test. (C) A diagram showing the height difference between the dividing wall and the mother anticlinal walls. (D) Plotting the height difference of the new cell wall (blue) and the average mother anticlinal wall (magenda), against new cell wall age. The height difference is estimated from the contact point maps of the AFM measurements (Materials and Methods) (*M. polymorpha* gemmae p-values: <6HAD - 20-30HAD= 0.013, 20-30HAD - second division= 0.0023. *A. thaliana* leaf p-values: <6HAD - 20-30HAD= 0.0019. All p-values are computed as Wilcoxon Rank Sum Test). Note that the cells undergoing AFM testing are plasmolysed. Hence, this analysis illustrates a trend, and the specific quantitative values obtained are not directly comparable to physiological conditions.

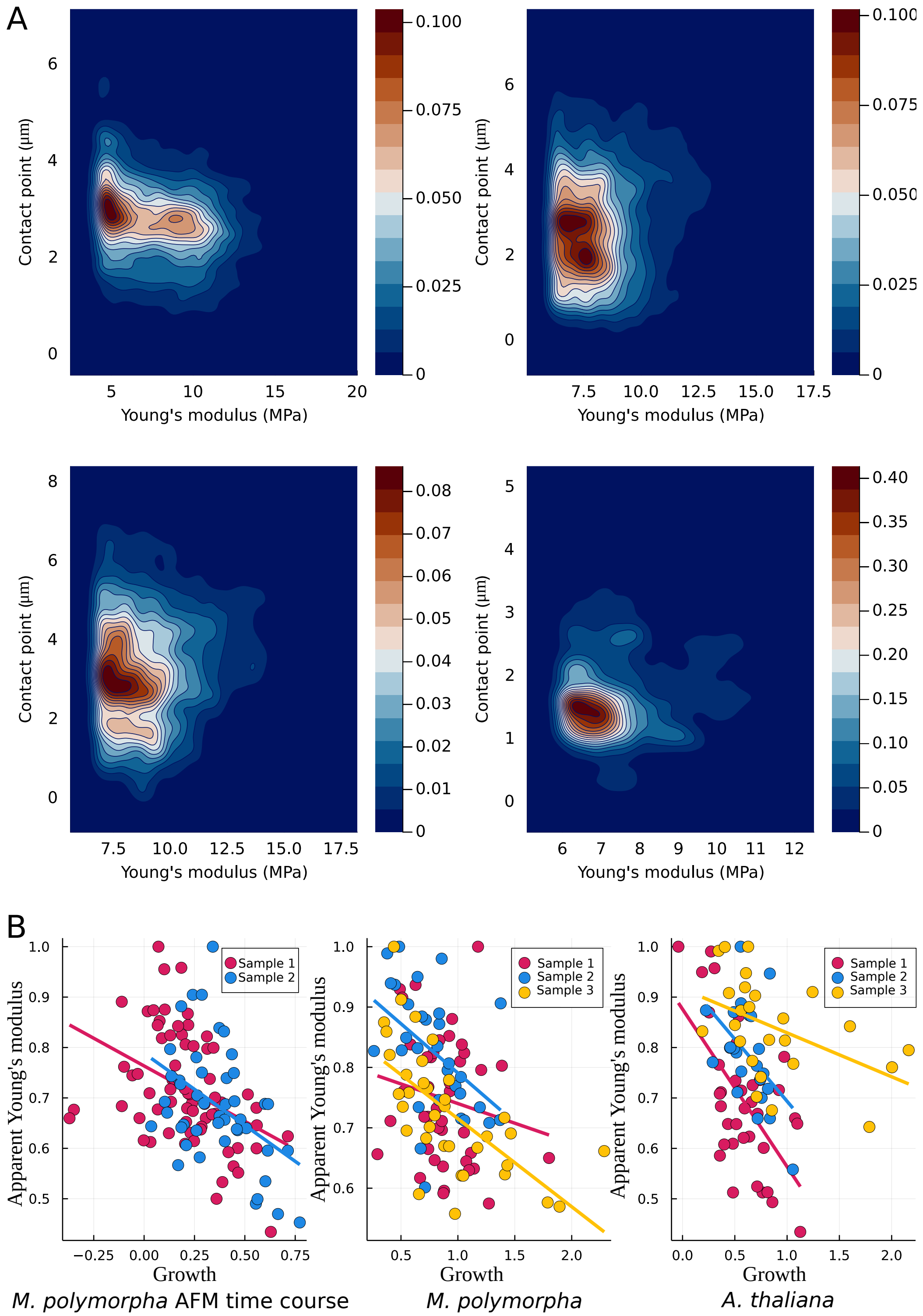

Fig. S6. (A) No correlation between height and Young's modulus is observed in AFM maps - 4 independent patches of cells from *M. polymorpha* gemmae. The plots refer to anticlinal walls only. These plots suggest that the stiffer new cell walls in *M. polymorpha* are not correlated to the fact that new cells might have a lower curvature. (B) Correlation between Apparent Young's modulus and growth for the *M. polymorpha* samples that underwent two AFM tests (AFM time course), for *M. polymorpha* gemmae, and for *A. thaliana* leaves.

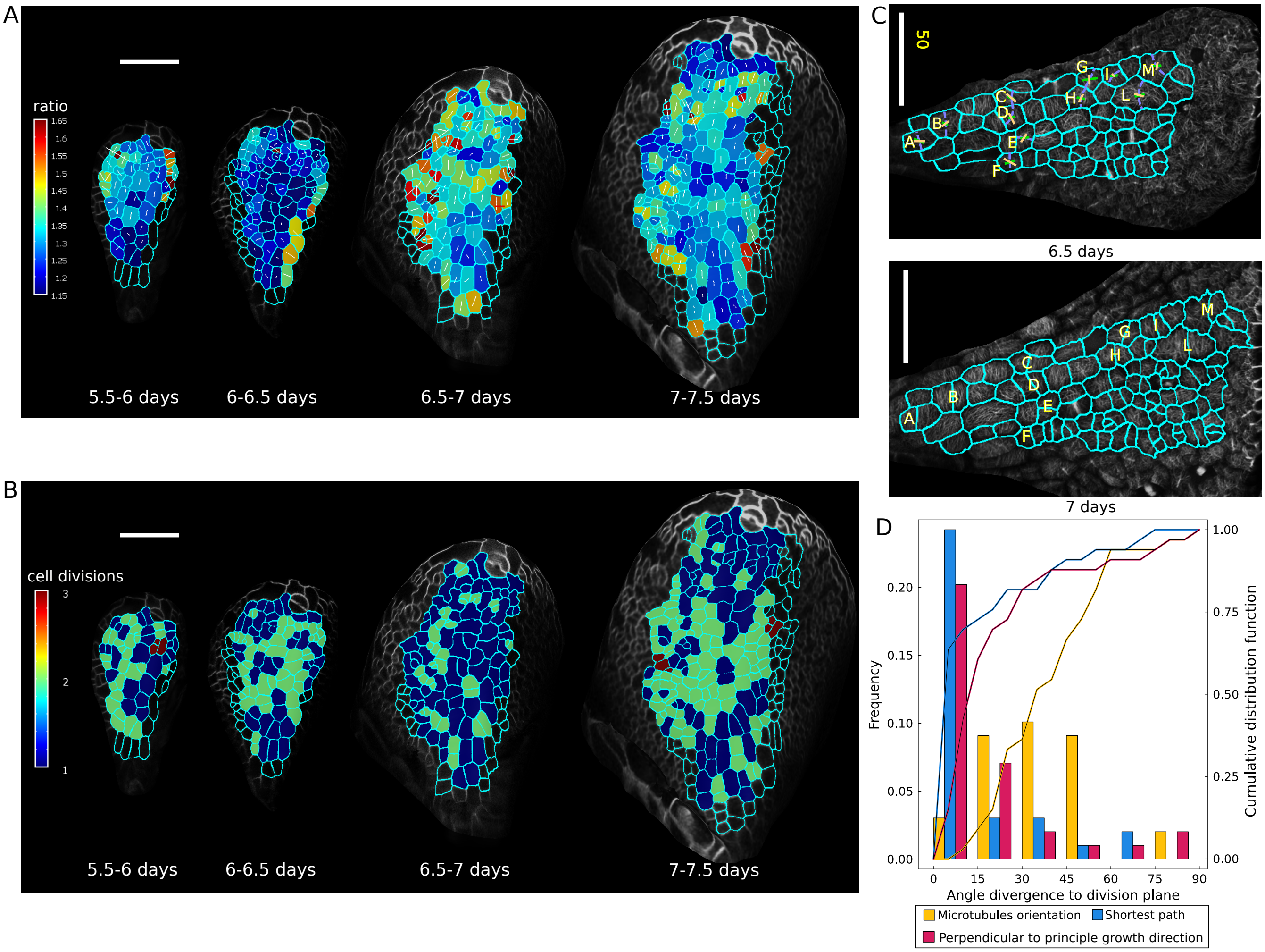

Fig. S7. Cell division in *A. thaliana* first true leaf follows geometrical clues. (A) Heat map of the ratio of cell area over the 12 hour time period shown in days after stratification, scale bar 100 μm. White lines show the major axis of expansion. (B) Heatmap of the number of cell divisions in the period of time shown, scale bar 100 μm. (C) Sequential imaging of cortical microtubules (MT) for extraction of cell shapes and MT orientation of an active cell division area, scale bar 50 μm. Maximum projection of cortical MT signal on extracted cell shapes shows the main MT orientation (yellow lines on left picture), while comparison of cell shapes between the two different time points allows the calculation of the maximum growth direction (green lines on left picture). Analysis of the cell shapes allows calculation of the shortest path connecting two non-consecutive cell walls (blue lines on left picture). Comparison of the MT main orientation and shortest path direction with the actual plane of division for the dividing cells (see same letters between top and bottom picture) shows that shortest path orientation predicts the orientation of the division plane. (D) Quantification of angles between the MT main orientation and the actual division orientation (yellow distribution), and of the angles between the shortest path and the actual division orientation (blue distribution), and of the angles between the Principle Growth Direction and the actual division orientation (pink distribution) for the cells for which the MT orientation and shortest path do not coincide with the division plane (cells for which both MT orientation and shortest path possess an angle of divergence to division plane < 15 have been removed), total number of cells 86. The yellow, blue and pink solid lines represent respectively the cumulative distribution for the three predictions–MT orientation, shortest path and perpendicular direction to PGD. A comparison of the cumulative distributions for the MT orientation and the shortest path shows that cell division in *A. thaliana* better follows the shortest path (Anderson-Darling test, p-value <0.0001).

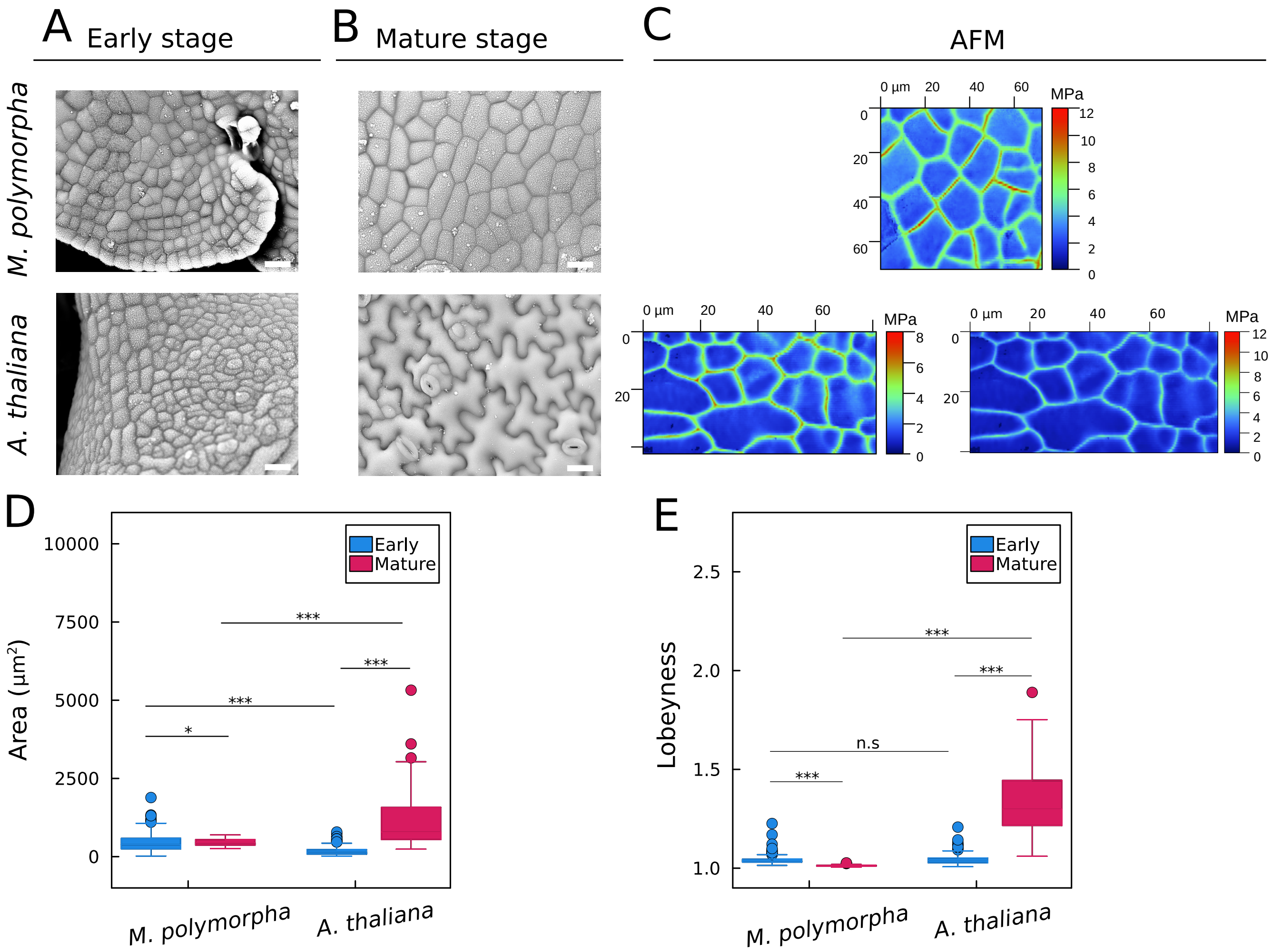

Fig. S8. Comparison of cell shapes at early (A) and mature - 3 weeks old (B) developmental stages for both species - *M. polymorpha* and *A. thaliana*. (C) Young's modulus of a patch of cells at an early developmental stage for the two species. For *A. thaliana*, the AFM map has been plotted with its optimised colour bar (image on the left) and with the same colour bar as *M. polymorpha* (image on the right). The latter map highlights the characteristic of *M. polymorpha* being stiffer than *A. thaliana*. (D) Cell area and (E) cell lobeyness for the two species at early and late stages of development (*M. polymorpha*: n_{early}= 294, n_{mature}= 57; *A. thaliana*: n_{early}= 302, n_{mature}= 110. The p-values are all < 0.0001, except the comparison between the area for *M. polymorpha* at early and mature stage p-value= 0.02, and the lobeyness at early stage between *M. polymorpha* and *A. thaliana* that results non significant). All p-values are computed as Wilcoxon Rank Sum Test.

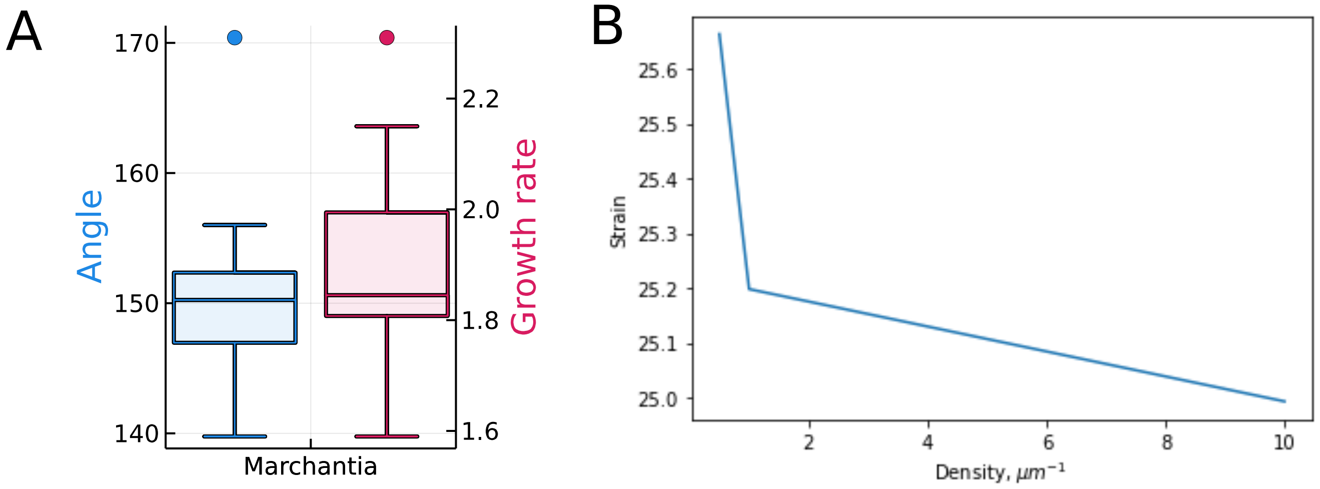

Fig. S9. Modelling details. (A) Quantification of the pinch-in angle 24 HAD in *M. polymorpha* gemmae and quantification of the cell growth area of the dividing cells 24 HAD. (B) Mesh convergence study performed on a regular hexagonal cell with increasing mesh density.

Methods

Immunostaining and image analysis of Marchantia Gemmae. Immunofluorescence of cell wall epitopes was performed as previously described (Alonso-Serra et al., 2020) with slight modifications. Four 48h old gemmae were fixed in a solution consisting of 4% formaldehyde (freshly prepared from paraformaldehyde powder, Sigma) and 0,5% glutaraldehyde (Sigma) in a 0.1M phosphate buffer at pH 7. Fixation, dehydration and resin infiltration steps were all micro-wave (MW) assisted using a PELCO BioWave Pro (Ted Pella, Redding, CA). Fixation was realized at 150W, under vacuum (20Hg) (5x 1'). Samples were left in the fixative overnight at 4°C and then washed 3 times in PBS. Samples were then processed through increasing dehydration steps (25%, 50%, 70%, 90%, 96%, 3x 100% Ethanol, vacuum 20Hg, MW 150W 5'). Resin infiltration (LR White medium grade, Agar scientific) was then realized through increasing resin concentration: 33% Resin in ethanol 100%, 66% Resin in ethanol 100%, and 3 times 100% Resin (20Hg, MW 200W 5'). Samples were left overnight in 100% resin for effective resin penetration in the samples. Samples were then aligned parallelly prior to resin polymerization, which was realized at 60°C during 17h. 1 µm thick sections were cut using an EM UC7 ultramicrotome (Leica Microsystems), mounted on Menzel Ultrafrost Plus microscopy slides on water droplets and air dried. Mounted samples were blocked with blocking solution (2% BSA in PBS supplemented with 0.05% Tween 20) for 30 min at room temperature. Primary antibodies LM19, LM20, LM12, LM15, LM25, LM23 and LM21 (Plant Probes, Leeds, UK) were applied as 1/100 dilutions in blocking solution over night at 4° C and slides washed 5 times in blocking solution. Alexa Fluor 488-conjugated Goat anti-Rat IgM (ThermoFisher Scientific, A-21212) secondary antibodies were as 1/200 dilutions in blocking solution for 2 h at room temperature and washed 5 times in blocking solution. For callose immunolocalization, callose primary antibody (Biosupplies) was applied in 500 µl as 1/400 dilution and secondary antibody Alexa Fluor 555-conjugated Goat anti-Mouse (ThermoFisher Scientific, A21425) secondary antibodies were applied as 1/200 dilutions in blocking solution for 2 at room temperature and washed 5 times in blocking solution. Slides were finally mounted in a 1:1 solution of AF1 antifadent (Citifluor) with PBS containing 0.1% (w/v) Calcofluor White (Merck/Sigma, F3543) as a cell wall counterstaining and then imaged by confocal laser scanning microscopy (Zeiss LSM700) and slides were sealed with nail varnish. Sections were subsequently imaged by confocal laser scanning microscopy as z-stacks (Zeiss LSM700).Fluorescence analysis of callose immunolocalizations was performed using a custom-made macro on ImageJ software. The sequential steps of the macro are as follows: images, acquired as z-stacks were first processed to maximal projections; a region of interest restricted to the cell division area is first hand drawn by the user; within the defined cell division region of interest, a binary mask is applied with a user defined threshold to the cell wall counterstaining channel (here calcofluor) in order to create a region of interest (ROI) restricted to cell walls in the cell division area (the creation of a region of interest restricted to the cell wall avoid to underestimate the levels of fluorescence measured in different conditions, as the studied epitopes are exclusively located to the cell wall); a second region of interest representing the older cell walls is defined as the inverse of the cell division area, and restricted to the cell walls using again a binary mask to restrict the ROI to the cell wall counterstaining; defined ROIs are finally applied to the immunolocalized channels, to measure the average fluorescence levels. The detailed macro code can be consulted in the github repository (3).

Statistical analysis of immunostaining experiments. Data analysis used the Tidyverse R package collection and the ggplot2 package for boxplots and barplots. Statistical analysis was performed using the R functions for ANOVA and Tukey HSD tests for multiple comparisons after having determined that parametric tests were applicable using R functions for Bartlett and Shapiro test. Significance values for $P < 0.05$ were grouped using the agricolae package (with alpha = 0.05). In the case parametric tests could not be applied, statistical analysis was performed using the R functions for Kruskal–Wallis one-way

analysis of variance and a non-parametric test for multiple comparison (nparcomp package) .

Modelling. Hexagonal shaped cells were generated by defining the nodes' positions and connections. The 3D meshes of the generated hexagonal outlines were obtained using a custom code (4) a Delaunay-based unstructured mesh generation algorithm from Darren Engwirda (5). All meshes were created so that the triangle edges on the cell boundaries were approximately 0.5 μm long, where they smoothly increase in size as they move away from the boundary to reduce the degrees of freedom in the system. This mesh was then converted into a compatible form to inflate in Tissue, (1, 6, 7) software developed by the Jönsson group.

Tissue uses a finite element method approach to approximate solutions to the hyperelastic continuous mechanical equations over the discretized mesh surface, which are being stretched by a normal (outward from each cell) pressure force applied to each mesh triangle. It uses the 'Triangular Bi-Quadratic Springs' (TRSB) method, which uses a St Venant Kirchoff formulation and approximates it using biquadratic springs, which resist triangle edge and inner angle deformation (8) and allows us to simulate cell walls efficiently. In more detail, the St Venant Kirchoff formulation stress-strain relation for an isotropic material is,

$$W_\Omega = \int_\Omega \left(\frac{\lambda}{2} (\mathrm{tr}\mathbf{E})^2 + \frac{\mu}{2} \mathrm{tr}\mathbf{E}^2 \right) d\Omega$$

where W is the total strain energy in a domain Ω, $\mathbf{E}$ the Green-Lagrange strain tensor and λ and μ are the Lame coefficients and defined in plane elasticity as $\lambda = \frac{E\nu}{1-\nu^2}$ and $\mu = \frac{E(1-\nu)}{1-\nu^2}$ with E being the Young's modulus and ν the Poisson coefficient. Using TRBS, W is approximated over a triangle T as

$$W_T = \sum_{i=1}^{3} \frac{k_i^T}{4} \left(L_i^2 - L_{i,0}^2 \right)^2 + \sum_{i \neq j} \frac{c_k^T}{4} \left(L_i^2 - L_{i,0}^2 \right) \left(L_j^2 - L_{j,0}^2 \right)$$

where k_i^T and c_k^T are the tensile and angular stiffness of the biquadratic springs and L_i and $L_{i,0}$ are the current and resting lengths of edge i in the triangle. These stiffnesses are defined as

$$\begin{aligned} k_i^T &= \frac{E\left(2\cot^2\alpha_i + 1 - \nu\right)}{16(1-\nu^2)A_T} \\ c_i^T &= \frac{E\left(2\cot^2\alpha_i \cot^2\alpha_j + \nu - 1\right)}{16(1-\nu^2)A_T} \end{aligned}$$

where α_i is the angle opposite edge i in the resting triangle configuration and A_T the resting triangle area. A force on each node can then be calculated from this strain energy as a result of the stretching triangles. Additionally, deriving the stress tensor for each triangle allows us to find the maximum stress magnitude and direction from the maximum corresponding eigenvector and eigenvalue.

The pressure force is applied to all the triangles proportional to their area in the outward normal direction. A solution to the simulation is found when the system has reached mechanical equilibrium, i.e. when the pressure forces on each node acting on them from their surrounding triangles are balanced with their surrounding stretched and strained triangles, such that the total force is now 0. We find this mechanical equilibrium using the Newton-Raphson method (9) and setting the tolerance to $1e-6$.

The hexagons' growth simulations were performed by first inflating to mechanical equilibrium and saving their state. The cross wall of interest was then given a higher stiffness and lower growth rate to replicate the effect of a higher apparent young modulus from the AFM data. The system was then allowed to grow using a spring with a variable resting length, a formulation previously introduced to model plant growth (10, 11). The resting length $L_{i,0}^t$ of the element i at time $t + \delta t$ with time step (δt) is given by the resting length in the previous time step $L_{i,0}^t$, plus the increment that is proportional to their strain and their current length. This formulation takes the form (1, 12)

$$L_{i,0}^{t+\delta t} = L_{i,0}^t + g_i \delta t \left(\frac{L_i}{L_{i,0}^t} - 1 \right) L_i$$

where g_i is the ~~growth rate of that~~ extensibility rate edge and L_i is the current edge length.

All simulations were inflated with a turgor pressure of 0.2 MPa, Young's modulus of 100 MPa and a Poisson's ratio of 0.2 and the growing hexagons had a extensibility rate of 0.2 hr^{-1}. The values chosen for the turgor pressure and the walls Young's modulus are in line with previous studies (13, 14). The stiffer cell wall has a extensibility rate of 0.1 hr^{-1} and a Young's modulus of 140 MPa when we vary both the extensibility rate and stiffness with the third contrapositive simulation having the

same parameters but just reversed. In the second simulation where we vary just the stiffness, the stiffer cell wall has a Young's modulus of 500 MPa (Table S4).

The simulations depicted in Figure 4A-D were stopped when the cells of interest (the one with the different cell wall properties); area, A satisfies

$$\frac{A - A_0}{A_0} > 2$$

where A_0 is the area of the cell at the beginning of the simulation to match with when the angles in Figure 4(G) were measured.

The black lines depicting the cell boundaries in Figure 4A-D were found using the indicator labels stored for each triangle in our mesh letting us know whether it starts the simulation on top of the cell or not (on the periclinal surface or not). From this we can then find the edge elements which are between one triangle on the top (the periclinal wall) and another on the side (the anticlinal wall).

References

1. O Hamant, et al., Developmental patterning by mechanical signals in arabidopsis. Science **322**, 1650–1655 (2008).
2. BE Shapiro, C Tobin, E Mjolsness, EM Meyerowitz, Analysis of cell division patterns in the arabidopsis shoot apical meristem. Proc. Natl. Acad. Sci. **112**, 4815–4820 (2015).
3. A Bonfanti, Plant cell division and growth (https://github.com/alebonfanti/plant-cell-division-growth) (2023).
4. (https://gitlab.developers.cam.ac.uk/slcu/teamsr/meshing_code) (2023).
5. D Engwirda, Locally optimal delaunay-refinement and optimisation-based mesh generation. (2014).
6. N Bhatia, et al., Auxin acts through monopteros to regulate plant cell polarity and pattern phyllotaxis. Curr. Biol. **26**, 3202–3208 (2016).
7. B Bozorg, P Krupinski, H Jönsson, Stress and strain provide positional and directional cues in development. PLoS computational biology **10**, e1003410 (2014).
8. H Delingette, Triangular springs for modeling nonlinear membranes. IEEE transactions on visualization computer graphics **14**, 329–341 (2008).
9. WH Press, SA Teukolsky, WT Vetterling, BP Flannery, Numerical recipes 3rd edition: The art of scientific computing. (Cambridge university press), (2007).
10. B Bozorg, P Krupinski, H Jönsson, A continuous growth model for plant tissue. Phys. Biol. **13**, 065002 (2016).
11. ET Smithers, J Luo, RJ Dyson, Mathematical principles and models of plant growth mechanics: from cell wall dynamics to tissue morphogenesis. J. Exp. Bot. **70**, 3587–3600 (2019).
12. A Armezzani, et al., Transcriptional induction of cell wall remodelling genes is coupled to microtubule-driven growth isotropy at the shoot apex in arabidopsis. Development **145**, dev162255 (2018).
13. A Sapala, et al., Why plants make puzzle cells, and how their shape emerges. Elife **7**, e32794 (2018).
14. RC Eng, et al., Katanin and clasp function at different spatial scales to mediate microtubule response to mechanical stress in arabidopsis cotyledons. Curr. Biol. **31**, 3262–3274 (2021).
